# Hyperchaos and the fusion of Moore’s automaton with gold sequences for augmented medical image encryption

**DOI:** 10.1038/s41598-025-29296-5

**Published:** 2025-12-12

**Authors:** Mohamed Gabr, Eyad Mamdouh, Dina El-Damak, Minar El-Aasser, Wassim Alexan, Amr Aboshousha

**Affiliations:** 1 Computer Science Department, Faculty of Media Engineering and Technology, German University in Cairo (GUC), New Cairo, 11835 Egypt; 2 Physics Department, Faculty of Basic Sciences, German University in Cairo (GUC), New Cairo, Egypt; 3 Electronics Department, Faculty of Information Engineering and Technology, German University in Cairo (GUC), New Cairo, 11835 Egypt; 4 Networking Department, Faculty of Information Engineering and Technology, German University in Cairo (GUC), New Cairo, 11835 Egypt; 5 Communications Department, Faculty of Information Engineering and Technology, German University in Cairo (GUC), New Cairo, Egypt; 6 Physics Department, Science Faculty, Cairo University, Giza, 12613 Egypt

**Keywords:** Augmented image encryption, Color image encryption, Differential equations, Entropy, Gold sequences, Medical image encryption, Memristive Coupled Neural Network Model, Moore’s Automaton, Applied physics, Electrical and electronic engineering

## Abstract

This study presents a sophisticated encryption methodology specifically designed for the secure transfer of medical images across cloud services. The initial phase of the algorithm involves the consolidation of multiple images to form a single augmented image, which is then subjected to the first layer of encryption. This layer employs an encryption key and an S-box generated through a Memristive Coupled Neural Network Model (MCNNM), establishing a strong foundation for security. Following this, the novel integration of Moore’s Automaton with Gold sequences is applied as a confusion mechanism, intrinsically scrambling the image structure to effectively disrupt pixel correlations. The encryption process iterates over *N* cycles, significantly deepening the level of encryption with each iteration. Performance evaluations reflect a considerable key space of $2^{2020}$ and a high encryption rate of 15.5 Mbps, while rigorous statistical tests validate the algorithm’s resilience. The encryption system proposed in this manuscript not only ensures a formidable level of security but is also pragmatically designed for application in the protection of sensitive healthcare data.

## Introduction

Healthcare systems are high-value targets in the global threat ecosystem, where adversaries increasingly deploy ransomware, data exfiltration, and supply chain attacks that traverse cloud providers, PACS/VNA repositories, telemedicine platforms, and AI pipelines. The timeliness and significance of medical image encryption algorithms cannot be overstated in today’s ever-connected digital world, where healthcare information systems are increasingly becoming targets for cyber attacks^1^. With the medical sector’s growing reliance on telemedicine and electronic health records, protecting patient data, particularly sensitive imaging such as MRIs, CT scans, and X-rays, has become paramount^2,3^. The exchange of medical images over cloud data platforms occurs frequently during remote diagnostics and consultations, making the security of such transfers a critical concern. As medical images are transmitted across networks to and from cloud storage and through third-party tools used for analytics or AI-driven workflows, the points of data transfer and integration become potential vulnerabilities^4^. Medical image encryption algorithms serve as a critical barrier, safeguarding patient confidentiality and ensuring compliance with stringent privacy regulations like HIPAA (HIPAA is the Health Insurance Portability and Accountability Act, which is a United States legislation that provides data privacy and security provisions for safeguarding medical information.) and GDPR (GDPR is the General Data Protection Regulation, which is a regulation in EU law on data protection and privacy in the European Union and the European Economic Area. It also addresses the transfer of personal data outside the EU and EEA areas.). They are especially crucial when transferring patient images for remote diagnosis, flowing across PACS/VNA infrastructures and telemedicine platforms, or when storing them in centralized databases accessible via the cloud. This practice is becoming commonplace, as cloud-based solutions offer scalable storage and accessibility for telehealth services^5^. As cyber threats evolve, the development of robust, efficient, and adaptable encryption algorithms for medical imagery is essential for preventing unauthorized access and potential data breaches that could lead to misuse of personal health information, loss of public trust, and substantial legal repercussions^6^. Furthermore, in the era of big data and AI, these algorithms are vital for maintaining data integrity for medical research and diagnostics, ensuring that insights derived from medical imaging are based on untampered, authentic sources^7^.

The core problem is to design an encryption scheme that satisfies five coordinated requirements. First, it must ensure confidentiality and integrity while resisting modern cryptanalysis and traffic analysis^3^. Second, it must scale to batch and streaming workloads across cloud and edge with low latency and high throughput^8^. Third, it must disrupt pixel correlation and modality redundancy to blunt inference and differential attacks^9^. Fourth, it must enable selective encryption and integrate with medical standards to preserve clinical usability^10^. Fifth, it must resist key recovery and brute-force attempts through a large, well-structured key space^11,12^. From these points, one can formulate the following research question. How can a medical image encryption scheme be designed and implemented so that, within real clinical and cloud workflows, strong confidentiality and integrity against modern attacks are achieved, pixel correlation and modality redundancies are disrupted, selective encryption and standards-compliant interoperability are supported, batch and streaming scenarios are scaled with low latency and high throughput, and brute-force resistance is provided through a large, well-structured key space?

Classical encryption algorithms like AES, DES, 3DES, and Blowfish, while robust and widely used for general data security purposes, face specific challenges when applied to medical image encryption^13^. Medical images are typically large files that require significant computational resources to encrypt and decrypt using these traditional algorithms, potentially leading to inefficiencies in clinical workflows where rapid access to images is often crucial^14^. In light of these challenges, the practice of batch encryption, where multiple images are encrypted in a single operation, can offer considerable benefits. By processing images in batches, encryption systems can take advantage of economies of scale, as common computational tasks are streamlined, reducing the overall processing time compared to encrypting each image individually. This approach not only improves efficiency but also reduces the latency introduced when transmitting images over bandwidth-limited networks to remote locations for diagnosis or consultation^2^.

Moreover, classical encryption algorithms were not designed to handle the high redundancy and strong correlation between pixels found in medical images, which can lead to suboptimal encryption and potential vulnerabilities. Additionally, batch encryption can mitigate the issue of high redundancy in medical images, as it enables the use of more sophisticated algorithms that recognize and efficiently handle patterns across a set of images, rather than redundantly processing similar patterns in each image^8,15^. Furthermore, they do not support selective encryption efficiently, which is desirable in medical imaging to allow for quick previewing of non-sensitive parts of an image while keeping sensitive information secure. The batch processing of images can also be compatible with techniques of selective encryption, allowing for certain regions across a collection of images to be encrypted while others are left for immediate access; this can be particularly useful when dealing with series of images where only certain frames contain sensitive information^16^. Taken together, these limitations motivate a shift from general-purpose ciphers to batch-aware, imaging-specific approaches, whose performance implications are especially evident under real network and workflow constraints of the healthcare industry.

As healthcare providers often need to transmit images over bandwidth-limited networks to remote locations for diagnosis or consultation, classical algorithms can introduce latency that impedes timely medical decision-making. Batch encryption can contribute to solving this by reducing the data overhead in the encryption process and the number of separate encryption transactions required, thereby decreasing the total time taken for images to be securely transmitted and decrypted at the destination^17^. This advantage is critical in telemedicine applications where time efficiency directly correlates with patient outcomes^18^. Therefore, there is a growing need for specialized encryption techniques that can handle the unique requirements of medical image data both securely and efficiently^19^. The ability to encrypt multiple images at once can be a pivotal feature of these techniques, addressing the constraints of traditional encryption methods and supporting the dynamic needs of modern medical practices^20^.

The utilization of solutions derived from systems of hyperchaotic differential equations in developing image encryption algorithms provides a powerful toolkit for enhancing data security^11,21–24^. The complex dynamics of hyperchaotic systems, characterized by multiple positive Lyapunov exponents and a greater sensitivity to initial conditions, result in highly unpredictable behavior that is advantageous for encryption^25,26^. This unpredictability translates into an expansive key space and an intricate key generation mechanism, making brute-force attacks practically infeasible^27^. Additionally, the rich structure of hyperchaotic trajectories, with their rapid divergence and intricate folding patterns, ensures that even minute differences in initial conditions or system parameters yield vastly different outcomes, thereby enhancing the robustness of the encryption against differential attacks^28^. Implementing hyperchaotic solutions in image encryption algorithms allows for the scrambling and diffusion of pixel values in a way that is non-repetitive and highly sensitive to the encryption key, which is essential in protecting the integrity and confidentiality of sensitive imagery data, such as medical or personal photographs, against sophisticated cyber threats^29,30^.

Linear Feedback Shift Registers (LFSRs) are simple yet powerful digital circuits used extensively as pseudo-random number generators (PRNGs) due to their efficiency and ease of implementation in hardware. Their lightweight nature makes them particularly suitable for electronic devices with limited processing capabilities, where they are often physically integrated into silicon at the chip level to assist in various functions, including cryptography and error detection^31^. An LFSR generates a sequence of bits by shifting the bits in the register and performing a linear operation–typically an exclusive or (XOR)–on some of its bits, known as tap positions, determined by a feedback polynomial. The electronic design of LFSRs allows for high-speed operation and minimal power consumption, which is crucial for battery-operated devices such as mobile phones and medical sensors. The appeal of LFSRs in PRNG applications lies in their predictability and speed, which are particularly beneficial in environments where performance is critical. In image encryption applications, LFSRs can be employed to generate pseudo-random sequences for the scrambling of pixel positions or the alteration of pixel values, which, when done properly, enhances the security of the encryption algorithm^32^. The sequences produced by LFSRs, thanks to their well-chosen feedback polynomials, exhibit desirable properties such as a long period and good statistical distribution. These properties are highly valued in the design of electronic communication systems where data integrity and noise immunity are paramount. However, it’s important to note that by themselves, LFSRs are not secure enough for high-security applications due to their linear nature, which can be susceptible to cryptanalysis. Consequently, in electronic security applications, LFSRs are often combined with non-linear operations, such as substitution boxes (S-boxes), or used as components in more complex encryption algorithms to achieve a higher level of security, suitable for protecting image data^33^.

A Moore automaton is a finite state machine whose output depends only on the current state, not directly on the input. It consists of states, input and output alphabets, a transition function from state–input pairs to next states, an output function from states to outputs, and an initial state. Because outputs are state-based, they change only after a state transition, creating a one-step latency relative to input changes. This simplifies synchronous circuit design and protocol control, though it can require more states than a Mealy machine. Recent literature on image encryption has thus seen its application^34^.

In medical image encryption algorithms, S-boxes are of paramount importance due to their ability to introduce non-linearity and significantly enhance the security level of the encryption process^35^. Medical images often contain highly sensitive and personal information, and their protection is critical to maintaining patient privacy and adhering to regulatory compliance standards. The S-boxes contribute to the confusion aspect of encryption, effectively disrupting the correlations and predictable patterns within medical images. This is particularly vital given the structured and redundant nature of medical imaging data, where pixel values often exhibit less variability than in other types of digital images (consider grayscale DICOM medical images, for example^2^). The robust transformation provided by S-boxes ensures that even minor differences in pixel values are amplified, leading to substantial changes in the encrypted output. Consequently, this makes it exceedingly difficult for unauthorized entities to reverse-engineer the encryption or to obtain any meaningful data without the correct decryption key, thus safeguarding sensitive medical information against unauthorized access and potential data breaches^36^.

The concepts explored in the previous discourse have catalyzed the conception and advancement of the enhanced medical image encryption algorithm proposed in this study. The primary contributions can be summarized as follows.

1. Employing a hyperchaotic system of differential equations that is representative of a neural network for medical image encryption is an innovative approach, particularly when combined with Moore’s automaton and Gold sequences. This blend of mathematics, physics, computer science, and electrical engineering principles yields a potent medical image encryption tool aligned with Shannon’s principles of secure communication^37^.
2. The utilization of three stages of encryption equips the proposed algorithm with a high resistivity against a wide range of cryptanalysis attacks, unlike some recent works in recent literature^38–44^.
3. The proposed algorithm demonstrates a strong resistance to brute-force attacks, achieving an expansive key space of $2^{2020}$ that provides robust security against such invasive attempts.
4. The combination of parallel processing techniques with batch-encryption results in a high level of efficiency, achieving an average encryption rate of 15.5 Mbps when processing medical images.
5. The proposed algorithm successfully meets all the criteria of the NIST SP 800-22 suite of tests, confirming that the output encrypted medical images possess the attribute of randomness.

The structure of this paper is organized as follows. Related literature depicting the latest research works on image encryption are surveyed in Sect. “Related literature”. Foundational concepts that underpin the proposed image encryption algorithm are introduced in Sect. “Preliminary constructs”. The procedural framework of the implemented encryption technique is detailed in Sect. "Proposed augmented medical image cryptosystem". An assessment of various performance metrics is presented, alongside a comparative analysis with existing methodologies from the literature, in Sect. “Performance evaluation”. The paper is concluded in Sect. "Conclusions and future outlook", where findings are summarized and potential avenues for prospective developments are indicated.

## Related literature

The quest for robust image encryption techniques has been an area of intense research, reflecting the increasing need to secure visual data in the digital age. This section delves into a spectrum of methodologies that have been recently explored, setting the stage for understanding the context and significance of the contributions made by the proposed augmented medical image encryption algorithm.

The authors of^34^ propose an innovative approach to image encryption systems, utilizing an Escalation function in conjunction with a unique, single-use key-dependent Moore’s Automaton defined over the binary finite field $\mathbb {F}_2$. The Escalation function employed in their work is a non-linear method that shuffles plaintext images during the confusion stage, playing a pivotal role in the permutation of rows and columns. To enhance the security and strength of the algorithm during the diffusion process, the utilized Moore’s Automaton generates encrypted images via a key stream. This key stream is highly randomized, produced by merging a Logistic map with a cyclic group. In particular, the Moore’s Automaton in question operates on $\delta (q_k)/\mathbb {F}_2$, which is instrumental in converting random bits into sequences that are difficult to predict, thereby forming secure ciphertext images.

A high-performance 5-stage image cryptosystem is implemented by the authors of^45^ using a mix of Arnold’s Cat map, the Mersenne Twister (MT), Langton’s ant, and S-boxes. The main idea of Langton’s Ant is using an ant or multiple ants that can step on a large array of squares and can turn right or left based on the color of the square. A Langton’s ant behaves in chaotic form for a certain number of steps (approximately 10000 steps for a single ant). $256 \times 256$ Composite Ant Fields (CAF) can be generated according to the number of sub-fields, the number of ants in each sub-field, the starting position of each ant in the sub-field, the starting direction of each ant, the number of unique colors each square in the field can have, and the rules followed by every ant to turn right or left according to the color of the square. The generated CAFs are employed as encryption keys for the 3 color channels in the first layer of the cryptosystem. The Mersenne Twister pseudo-random number generator (PRNG) is used for obtaining S-boxes and encryption keys for the second, third, and fifth layers. The Mersenne Twister is accessible to many developers using Wolfram Mathematica^®^. Finally, Arnold’s cat map shuffles the image pixels multiple times in the fourth layer according to the following transformation:

1 $$\begin{aligned} \begin{bmatrix} x_{n+1}\\ y_{n+1} \end{bmatrix} = \begin{bmatrix} 1 & a\\ b & 1+ab \end{bmatrix} \begin{bmatrix} x_n\\ y_n \end{bmatrix} (mod\textit{ N}), \end{aligned}$$

where *x* and *y* represent pixel coordinates, *N* represents the full size of the image, and *a* and *b* are input variables.

Using DNA coding, the Chen hyperchaotic system of fractional-order, the discrete Fourier transform (DFT), and the MT as a PRNG, the authors of^46^ created a 3-stage image cryptosystem. First, a $256 \times 256$ plain image is converted to a 1D DNA sequence. Next, DFT is applied to the solution of the hyperchaotic Chen system of fractional-order. The DFT is split into a real part $Re(F(Sol_{CK}))$ and an imaginary part $Im(F(Sol_{CK}))$. The system computes the median of the real and imaginary parts and utilizes the medians as thresholds to transfer both parts of the solutions into bit-streams. Those bit-streams are converted to a DNA base named $Key_{Chen}$. The 1D DNA sequence of the image is added to the $Key_{Chen}$ in the first layer. In the second stage, an S-box is formed using the solution of the hyperchaotic Chen system for data confusion. In the third stage, the output of the MT and the flattened processed image are converted to base-${\phi }$. Then, a modulo operation is performed to generate a sequence. This sequence is re-structured into the final encrypted image.

In^47^, the authors present a cryptosystem for medical images. Comparing the medical image to typical digital images, the former has smoother sections and less vivid shading and texture. The first step in their system is a matrix re-shaping of a plain image using the transpose function in Matlab^®^. The second step is the circular shift of the matrix elements by 1 position using the circshift function in Matlab^®^. Then, the Logistic-Tent map is utilized in the third step to encrypt the image further. The fourth step utilizes an Arnold’s transformation to scramble the image. Finally, the data is XORed with a pseudo-random number to generate the final encrypted image. The PRNG is implemented using a non-linear filter function-based linear feedback shift register (LFSR). A new 8-variable boolean function is designed and is shown to exhibit good cryptographic properties.

An image cryptosystem using the Affine Algorithm (AA), a LFSR, and XOR encryption is proposed by the authors of^48^. First, the pixel location of the RGB image is encrypted twice using AA and 2 keys for each encryption step. Second, the pixel values are encrypted using the AA using 6 keys (2 keys for each of the red (R), green (G), and blue (B) channels). Then, the 3 channels are combined to form the image. Third, 256 keys are generated using the LFSR. The XOR operation is employed to encrypt the image by first encrypting the rows and then encrypting the columns using the LFSR keys. The 3 steps are repeated in rounds to generate the final encrypted image.

By altering the pixels’ placement and encrypting the pixel values utilizing DNA coding and 3D chaotic maps, the authors of^49^ present an image cryptosystem. First, a color image is separated into its 3 RGB channels, and each channel is divided into 2 parts. Then, a LFSR generates a key that is used to redistribute the positions of the pixels in each part. Second, the values of the pixel are encrypted utilizing DNA coding. Third, the image is XORed with the key of the LFSR after further processing. Fourth, the data is encrypted by XORing each channel with a key generated using 3D chaotic maps. Finally, the RGB color channels are combined once again to form the output encrypted image.

The authors of^32^ present an image cryptosystem using a non-linear feedback shift register (NLFSR). The cryptosystem is made of 2 stages. The first is for pixel permutation to modify the locations of the pixels, and the second is for modifying pixel values. The first step is completed by shuffling the rows and columns using 2 pseudo-random number sequences generated by the NLFSR. Then the output of the first stage is converted to a 1D bit stream, encoded as a DNA sequence, and added to a third DNA-encoded pseudo-random number sequence generated by the NLFSR. The NLFSR is implemented in a Galois configuration with a feedback function applied to each of its states.

In^50^, the authors present a medical image cryptosystem using both confusion (pixel scrambling) and diffusion (pixel value encryption). First, confusion is accomplished by dividing the image into tiles, rearranging them using a zigzag algorithm, and rotating the tiles by 90 degrees. Finally, pixel scrambling is completed by the random permutation of the image tiles. The pixel value is encrypted by XORing the image after the confusion stage with a key generated using a chaotic map. The simple Logistic map is utilized in that work as the chaotic map of choice for implementing the diffusion stage.

## Preliminary constructs

### Memristive Coupled Neural Network Model

In^51^, Lin et al. developed the Memristive Coupled Neural Network Model (MCNNM), which is composed of smaller neural networks that are designed using the Hopfield Neural Network (HNN) framework along with a memristive system inspired by the flux-controlled memristor. This network harnesses the chaotic nature akin to that found in the brain’s neural system to replicate the chaotic activity observed in the nervous system of the brain. The mathematical representation of this model is as follows:

2 $$\begin{aligned} C_i\dot{v}_i=-\frac{v_i}{R_i} + \sum w_{ij}tanh(v_i) + I_i \quad \quad (i,j \in N^*) \end{aligned}$$

In the given context, $C_i, R_i,$ and $v_i$ signify the capacitance, resistance, and membrane potential of the neuron labeled *i*, respectively; $\dot{v}$ is the rate of change of a membrane’s potential during stimulation; $w_{ij}$ represents the synaptic weight coefficient that characterizes the strength of the connection from neuron *j* to neuron *i*. The stimulation of the neuron is depicted by the hyperbolic tangent function, while $I_i$ stands for the external input current. It’s important to highlight that the chaotic dynamics of the HNN are highly dependent on the synaptic weights $w_{ij}$. Consequently, 2 separate sub-neural networks each comprising 4 neurons can be constructed based on the inherent HNN structure in (2), by selecting appropriate synaptic weight coefficients through a trial-and-error process.

**Fig. 1:**
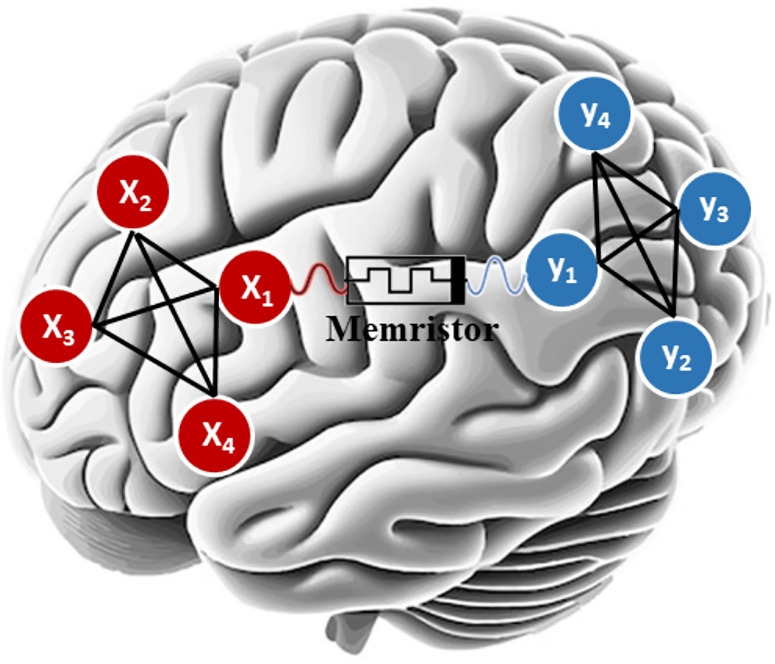
The connection of 2 sub-neural networks with a memristor^51^.

Figure 1 illustrates the interconnection of the 2 sub-neural networks via a memristor, where $X_i$ and $Y_i$ represent the 8 neurons involved. Therefore, under the assumption that $C_i = 1, R_i = 1, I_i = 0$ for $i\in {1,2,3,4}$, the MCNNM can be determined by the system of differential equations presented in (3):

3 $$\begin{aligned} \begin{aligned} \left\{ \begin{aligned}&\dot{x}_1=\beta _{1}x_1 +\beta _{2}tanh(x_1) + \beta _{3}tanh(x_2) - \beta _{4} tanh(x_3) \\ &\quad \quad -\beta _{5}tanh(x_4) + p\varphi (x_1 - y_1), \\&\dot{x}_2=\beta _{6}x_2 + \beta _{7}tanh(x_2) + \beta _{8}tanh(x_3) - \beta _{9} tanh(x_4), \\&\dot{x}_3=\beta _{10}x_3 + \beta _{11}tanh(x_1) - \beta _{12}tanh(x_2) + \beta _{13} tanh(x_3) \\ &\quad \quad +\beta _{14} tanh(x_4),\\&\dot{x}_4=\beta _{15}x_4 + \beta _{16}tanh(x_1) - \beta _{17}tanh(x_3) + \beta _{18}tanh(x_4),\\&\dot{y}_1=\beta _{19}y_1 + \beta _{20}tanh(y_1) + \beta _{21}tanh(y_2) - \beta _{22} tanh(y_3) \\ &\quad \quad - \beta _{23}tanh(y_4) -p\varphi (x_1 - y_1), \\&\dot{y}_2=\beta _{24}y_2 + \beta _{25}tanh(y_2) + \beta _{26}tanh(y_3) + \beta _{27} tanh(y_4),\\&\dot{y}_3=\beta _{28}y_3+ \beta _{29}tanh(y_1) - \beta _{30}tanh(y_2) + \beta _{31}tanh(y_3) \\ &\quad \quad - \beta _{32}tanh(y_4), \\&\dot{y}_4= \beta _{33}y_4+ \beta _{34}tanh(y_2) + \beta _{35}tanh(y_3) + \beta _{36}tanh(y_4), \\&\dot{\varphi }=\beta _{37}sin(\pi \varphi ) + (x_1 - y_1).\\ \end{aligned} \right. \end{aligned} \end{aligned}$$

where $\beta _{1}=-1$, $\beta _{2}=1.8$, $\beta _{3}=2$, $\beta _{4}=0.5$, $\beta _{5}=12$, $\beta _{6}=-1$, $\beta _{7}=1$, $\beta _{8}=20$, $\beta _{9}=0.5$, $\beta _{10}=-1$, $\beta _{11}=0.5$, $\beta _{12}=4$, $\beta _{13}=1.8$, $\beta _{14}=4$, $\beta _{15}=-1$, $\beta _{16}=0.82$, $\beta _{17}=0.5$, $\beta _{18}=2$, $\beta _{19}=-1$, $\beta _{20}=1$, $\beta _{21}=0.5$, $\beta _{22}=3.5$, $\beta _{23}=1$, $\beta _{24}=-1$, $\beta _{25}=2.8$, $\beta _{26}=3$, $\beta _{27}=0.5$, $\beta _{28}=-1$, $\beta _{29}=3$, $\beta _{30}=3$, $\beta _{31}=1$, $\beta _{32}=0.7$, $\beta _{33}=-1$, $\beta _{34}=0.5$, $\beta _{35}=1$, $\beta _{36}=1$, $\beta _{37}=1$. In this context, $x_i$ and $y_i$ denote the membrane potentials of neurons $X_i$ and $Y_i$, respectively; $\varphi$ represents the internal state variable related to flux; the term $p\varphi (x_1 - y_1)$ introduces a non-linear element that symbolizes the induction current between neighboring neurons $X_1$ and $Y_1$ when they have different membrane potentials; *p* signifies the coupling strength that influences the memristive magnetic induction effect, and $sin(\pi \varphi )$ indicates the additional magnetic flux that arises due to fluctuations in the membrane potential.

The chaoticity and fitness of the MCNNM system, as described in (3), are meticulously examined in^51^ through a variety of chaotic behavior analyses. These analyses include studies of dynamic behaviors related to the coupling strength, dynamic behaviors associated with initial conditions, as well as the presence of coexisting hyperchaotic attractors that are influenced by an initial boost.

The system in (3) can be numerically solved, in a manner similar to that carried out in^30^, by concatenating all the solutions obtained from all the $x_i$ in a single long list, then calculating their median value, $\lambda$. Next, every solution point is compared to $\lambda$, and turned into a 1 if it is larger than $\lambda$, or turned into a 0 otherwise. This allows for the generation of an encryption key in the form of a bit-stream from the system in (3). Recent works showcase similar usage of chaotic neurons to carry out multimedia security^52^.

### Moore’s automaton

A Moore automaton is a specific type of finite state machine (FSM), a fundamental concept in the field of computer science and automata theory. Named after Edward F. Moore, a Moore automaton is defined by a tuple consisting of a finite set of states, a start state, a set of input symbols, a transition function, and a set of output symbols^34^. Unlike the Mealy automaton where the outputs are determined by both the current state and the current input, in a Moore automaton, the outputs are exclusively determined by the current state. Each state in a Moore automaton is associated with a specific output value, and when the automaton enters a state, it produces the output corresponding to that state, regardless of the input that caused the transition. This characteristic leads to a simpler design and analysis of the system behavior, making Moore automata particularly useful in digital circuits and sequential logic design, where predictable, stable outputs are essential.

**Fig. 2:**
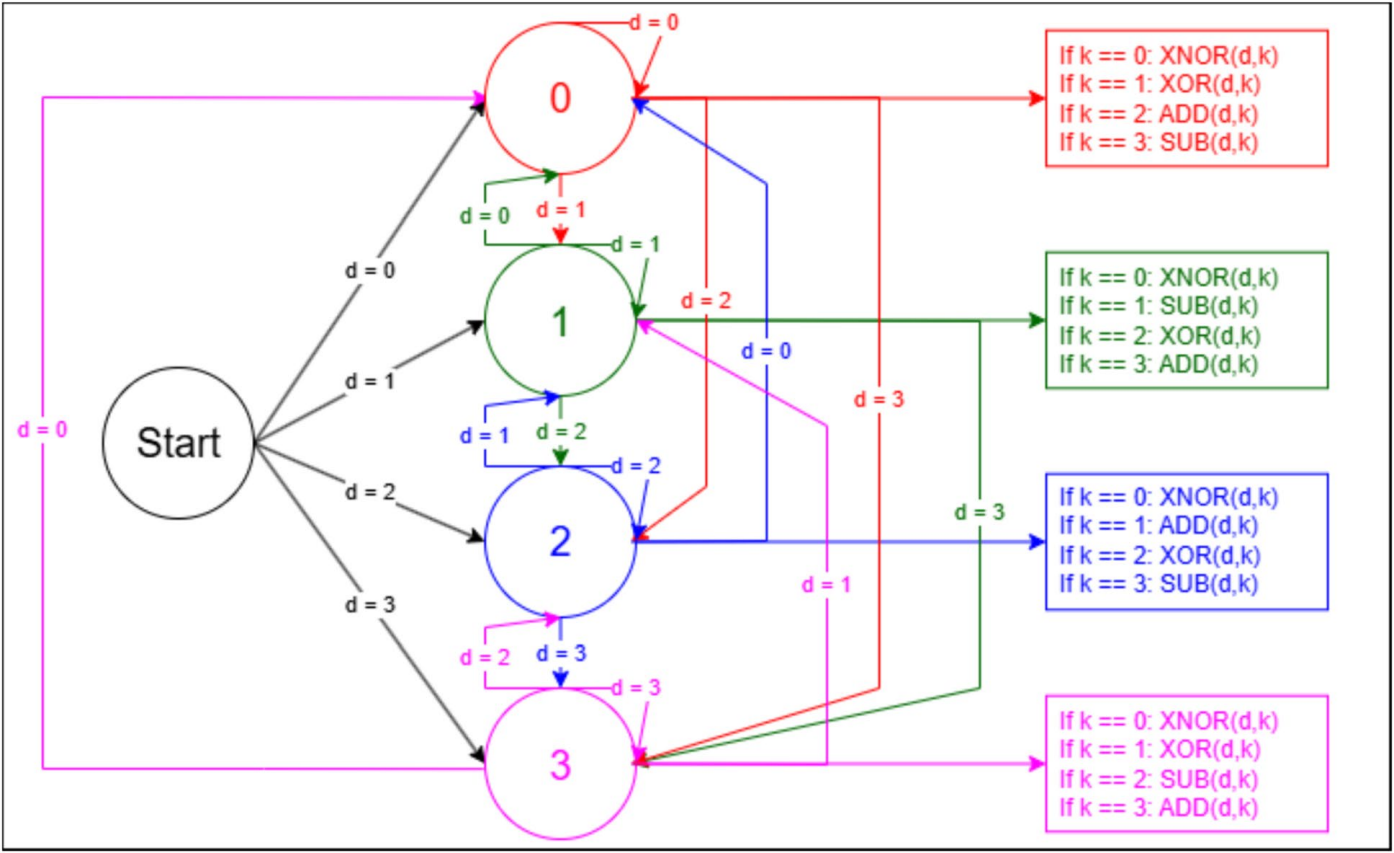
Full operation of the Moore’s automaton.

The main objective of Moore’s Automaton is to transform the image bits into a new data string. By taking the current image bits which is represented by *d* and *k* represents the current key bit. So, Moore’s Automaton takes the current image bit *d* and the current key bit *k* to decide which operation should be used. For example, if *d* is 0 and *k* is 1, this means that the current state is 0, and an XORing operation is carried out with the key **XOR(***d* **,** *k***)**. If the next image bit is 2 and the next key bit is 0, this means that the operation transforms from state 0 to state 2, and XNORing operation is carried out with the key **XNOR(***d* **,** *k***)**. This operation is visually illustrated in Fig. 2.

### Gold sequences

Gold sequences, named after Robert Gold, are a type of pseudo-random number generators (PRNGs) that are crucial in many digital communication systems. They belong to a larger family of sequences known as maximal length sequences or m-sequences, which are generated from linear feedback shift registers (LFSRs). What distinguishes Gold sequences is their optimal cross-correlation properties, which means that any two Gold sequences have a cross-correlation function that is tightly bounded^53^. This characteristic makes them highly resistant to interference and ideal for applications requiring synchronization and privacy, such as spread spectrum systems (including CDMA) and secure wireless communications^54^.

Their importance lies in their ability to maintain low cross-correlation with each other while being easy to generate, making them excellent candidates for channel coding, where multiple users share the same frequency space. By assigning a unique Gold sequence to each user, systems can efficiently differentiate between signals in multi-path environments, a common scenario in mobile and satellite communications. This property of Gold sequences, to remain distinct amid noise and interference, significantly enhances the reliability and fidelity of communication channels, ensuring data integrity and security in complex digital communication networks^55^.

**Fig. 3:**
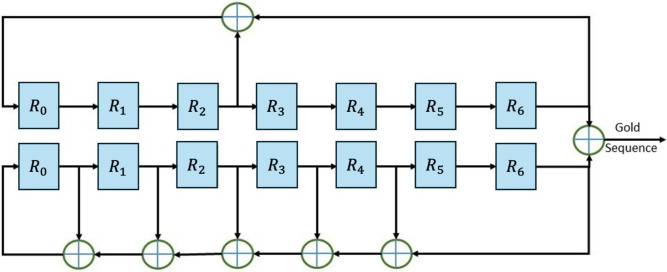
Flowchart of the gold sequence generator based on example generator polynomials $g_1(x)$ and $g_2(x)$.

Figure 3 depicts an example circuit that generates a Gold sequences of length $2^7-1$. The two sequence generator polynomials are modeled by $g_1(x)=x^7+x^3+1$ and $g_2(x)=x^7+x^5+x^4+x^3+x^2+1$. In Fig. 3, the $R_i$ represent flip-flops, each capable of storing and shifting a single bit at a time. It is clear that only simple circuitry is needed to generate a large number of unique codes, as long as the initial values of registers are not all zeros.

## Proposed augmented medical image cryptosystem

This section presents the structure of the proposed augmented medical image encryption algorithm, detailing the processes of augmented medical image formation, and the methods for encrypting and decrypting those augmented images. These components are described in dedicated subsections, specifically Subsection "Augmented medical image formation" for augmented medical image formation, Subsection “Encryption methodology” for the encryption methodology, and Subsection “Decryption methodology” for the decryption methodology.

### Augmented medical image formation

**Fig. 4:**
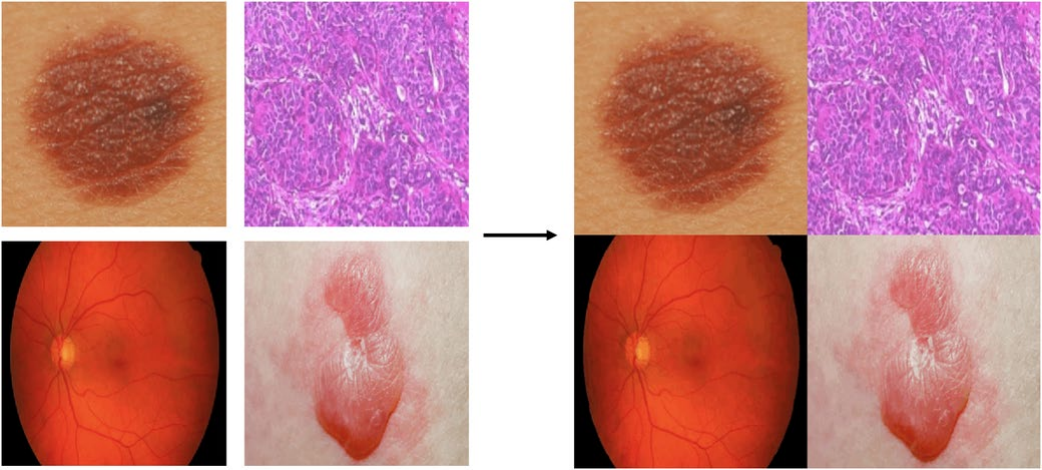
The formation procedure of an augmented medical image.

The proposed algorithm initiates with a preprocessing step that takes as input a number of medial images and results in an output of a single augmented image. Figure 4 provides a visual illustration of this step, with an example of 4 images as an input and their corresponding output augmented image of dimensions $512\times 512$ from 4 images, each of dimensions $256\times 256$.

### Encryption methodology

In this section, the construction of the encryption process is demonstrated as per the following steps:

1. Stage 1:
  - Starting by a set of input images, an image *I* is formed as the result of augmenting this set of image into a single augmented one.
  - Image *I* is then converted into a 1D bit-stream $I_{1D}$.
  - Given $Seed_{HNN}$, the HNN system is solved, producing a bit-stream $Key_{HNN}$ of a length $L_{HNN}$ which is shorter in length than $I_{1D}$.
  - Towards increasing the value of $L_{HNN}$, Algorithm 1 is invoked with the parameters $Key_{HNN}$, $Length(I_{1D})$, alongside $Seed_{prime}$.
  - $I_{1D}$ and the extended $Key_{HNN}$ are then XORed producing $I_{1D,HNN}$.
2. Stage 2:
  - Given $Seed_{GoldSBox}$, the Gold sequence is solved producing a bit-stream.
  - Algorithm 2 is invoked with the previously produced bit-stream resulting in generating $SBox_{Gold}$ (an example S-box is shown in Table 1).
  - $I_{1D,HNN}$ is transformed back into an image, then subjected to $SBox_{Gold}$, producing $I_{1D,HNN,SBGold}$.
3. Stage 3:
  - Given $Seed_{GoldKey}$, the Gold sequence is solved once more producing $Key_{Gold}$.
  - $I_{1D,HNN,SBGold}$ and $Key_{Gold}$ are then passed as parameters to the Moore’s automaton, producing $I_{1D,HNN,SBGold,MGold}$.
  - $I_{1D,HNN,SBGold,MGold}$ is transformed back into an image producing the augmented encrypted image $I'$.

**Fig. 5:**
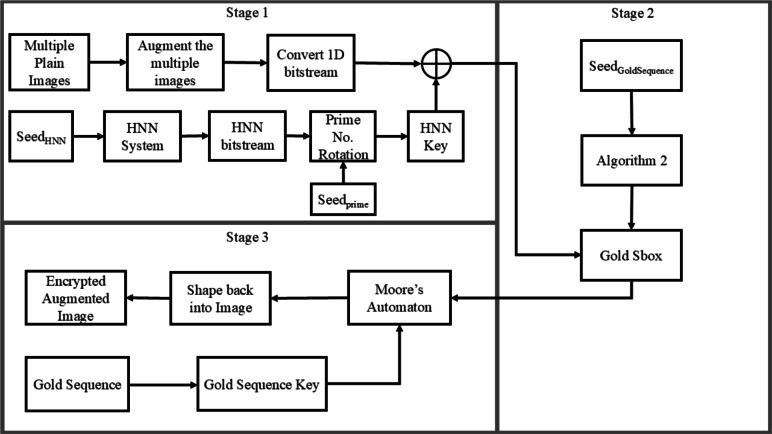
Encryption methodology flow chart, carried out *N* times.

A flowchart showing these steps is presented in Fig. 5.

**Algorithm 1:**
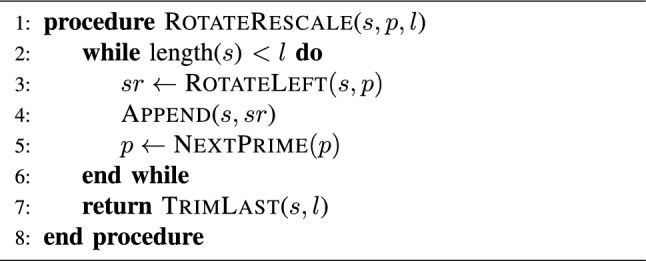
Prime Rotation Expansion for a Sequence *s*, Using Prime Seed *p*, to reach length *l*, (an adaptation from that proposed in^56^)

**Algorithm 2:**
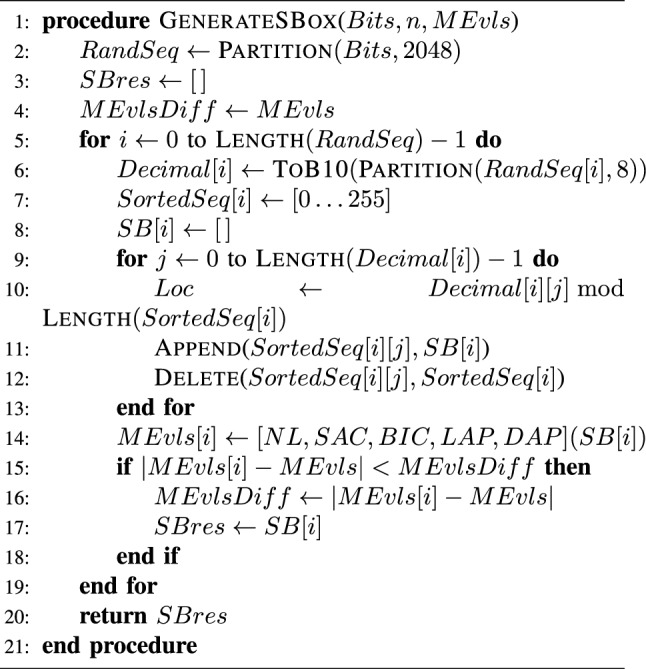
Construct an S-box given a bit-stream *Bits*, the number of S-box trials *n*, and target performance metrics $M_{Evls}=\{NL,SAC,BIC,LAP,DAP\}$ (an adaptation from that proposed in^27^)

**Table 1:** Gold sequence based S-box, based on a circuit with generator polynomials $g_1(x)=1+x^4+x^5+x^9$ and $g_2(x)=1+x^4+x^5+x^8+x^9+x^{10}$.

0	227	153	12	192	66	155	117	9	21	19	145	10	144	8	210
152	94	47	213	57	157	141	140	229	160	230	22	119	235	43	180
14	25	92	234	86	29	96	32	38	225	37	187	89	58	1	216
44	214	70	220	73	56	20	168	98	65	74	182	45	172	48	83
133	167	159	138	251	68	219	166	34	79	90	82	104	64	87	81
16	131	52	46	93	178	134	35	198	186	53	31	30	101	69	11
188	240	121	161	3	110	105	13	2	4	149	15	36	103	135	24
215	28	59	54	254	33	253	221	137	26	185	111	148	124	42	5
6	183	62	190	249	232	197	109	113	247	71	194	203	179	51	199
67	84	204	88	136	191	127	7	200	114	201	163	211	171	146	91
118	207	147	189	151	142	128	158	228	50	125	112	77	85	169	246
143	177	250	209	27	99	170	208	202	181	76	130	243	218	196	238
106	248	231	129	100	97	193	173	49	245	195	72	212	18	40	255
60	17	23	41	154	115	164	175	226	242	102	156	139	244	107	75
224	122	217	252	132	95	237	176	123	174	116	206	78	236	239	61
80	150	205	108	165	63	120	184	233	39	162	241	223	55	126	222

### Decryption methodology

As a reverse process to encryption, the decryption process is demonstrated as per the following steps:

1. Stage 3:
  - Image $I'$ is transformed into a 1D bit-stream $I_{1D,HNN,SBGold,MGold}$.
  - Given $Key_{Gold}$ (generated the same way as in the encryption process), and $I_{1D,HNN,SBGold,MGold}$, both are passed as parameters to the Moore’s automaton resulting in the production of $I_{1D,HNN,SBGold}$.
2. Stage 2:
  - The inverse of $SBox_{Gold}$, $SBox^\prime_{Gold}$ is generated.
  - $I_{1D,HNN,SBGold}$ is transformed back into an image, then subjected to $SBox^\prime_{Gold}$, producing $I_{1D,HNN}$.
3. Stage 1:
  - Given the extended $Key_{HNN}$, and $I_{1D,HNN}$, both are XORed producing $I_{1D}$.
  - $I_{1D}$ is transformed back into image *I*, which is further split into the set of plain images.

**Fig. 6:**
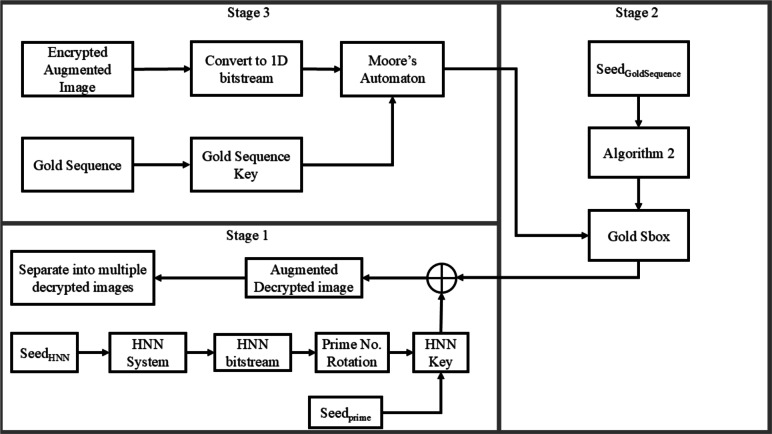
Decryption methodology flow chart, carried out *N* times.

A flowchart showing these steps is presented in Fig. 6.

## Performance evaluation

The security, efficiency and robustness of the proposed medical image encryption algorithm are meticulously assessed in this section. Two distinct sets of images have been employed for this evaluation: a) Medical images from^57^, which require stringent security measures to protect sensitive personal health information; and b) A selection of standard images from the USC-SIPI image database^58^, which is widely recognized for benchmarking purposes in the field of image processing. Through this approach, a comparative analysis with established algorithms in the scholarly literature is facilitated. Additionally, larger dimensioned augmented plain images are formed from smaller images of dimensions $256\times 256$ pixels and utilized to ensure that the algorithm’s performance is thoroughly vetted against a variety of complex scenarios, affirming its capability for practical encryption applications. Unless otherwise stated, the algorithm is carried out over a single iteration $(N=1)$. The software of choice on which the algorithm is implemented is Wolfram Mathematica^®^ v. 13.1, run on a machine having an AMD^®^ Ryzen 5 5600*H* CPU, running at 3.3 GHz, and having 16 GB of DDRAM.

A comparative analysis with a number of works from recent literature is carried out. These were selected based on the following criteria: (i) widely adopted classical or lightweight image ciphers that represent common design patterns in recent literature, (ii) chaos- and PRNG-based schemes that are architecturally closest to the proposed method, enabling like-for-like comparisons on diffusion, confusion, and key space, and (iii) works that publicly report the same evaluation metrics and image sets, which ensures reproducibility and fair comparison.

### Visual analysis

**Fig. 7:**
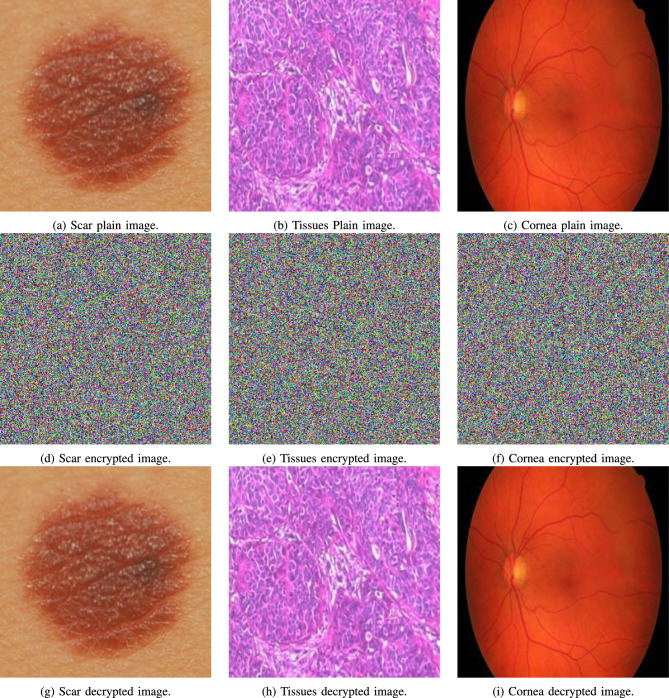
Visual analysis for various Medical images Scar, Tissues, and Cornea (**a**) to (**c**), as well as their encrypted and decrypted images (**d**) to (**i**), respectively.

**Fig. 8:**
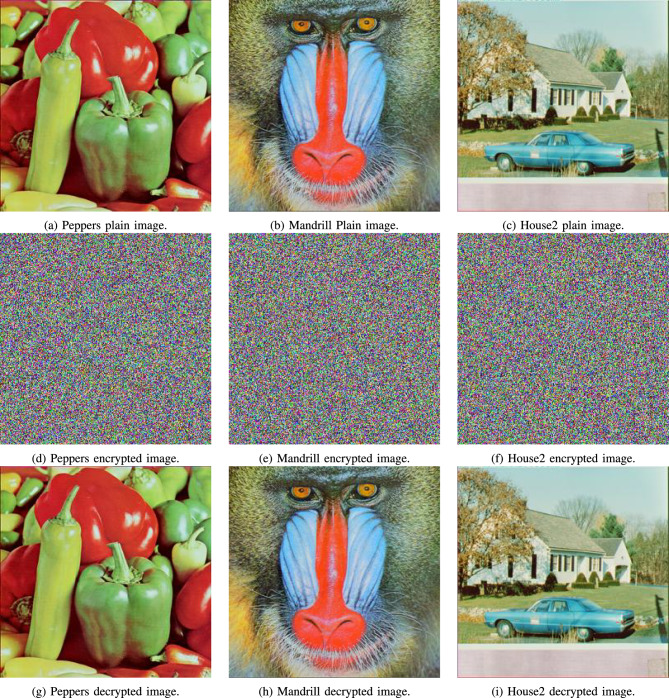
Visual analysis for various cover images Peppers, Mandrill, and House2 (**a**) to (**c**), as well as their encrypted and decrypted images (**d**) to (**i**), respectively.

Visual examination of the output encrypted images from the proposed algorithm confirms its encryption and decryption capabilities. Figs. 7 and 8 showcase both medical and non-medical images, as well as their encrypted and decrypted versions. It is clear that the encrypted versions of the images are noise-like and convey no information to their original plain counterparts. Furthermore, it is clear the decrypted versions are identical to their original plain versions, with no noise or artifacts added.

**Fig. 9:**
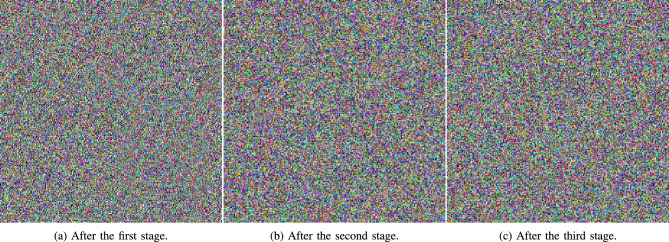
Encrypted images resulting after subsequent stages of encryption.

Moreover, Fig. 9 shows the effect of sequentially carrying out the encryption stages of the proposed algorithm.

### Histogram analysis

**Fig. 10:**
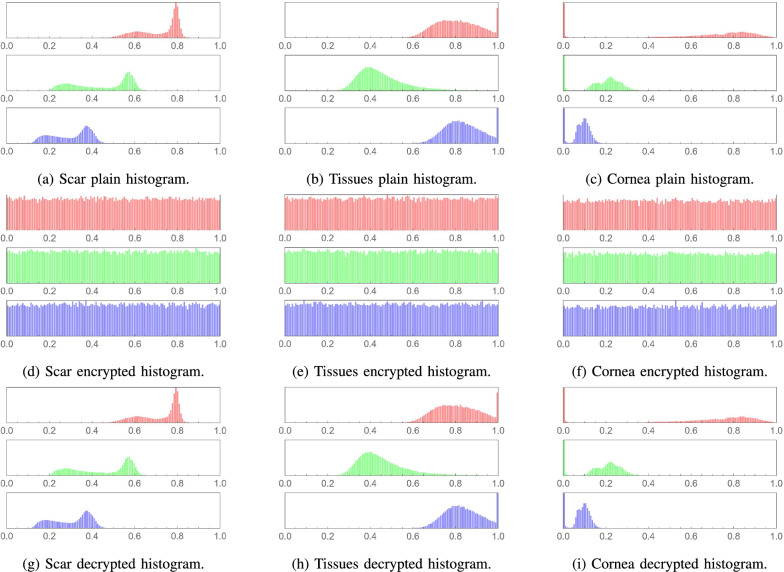
Histogram analysis for various medical plain images, their encrypted and decrypted versions, respectively.

**Fig. 11:**
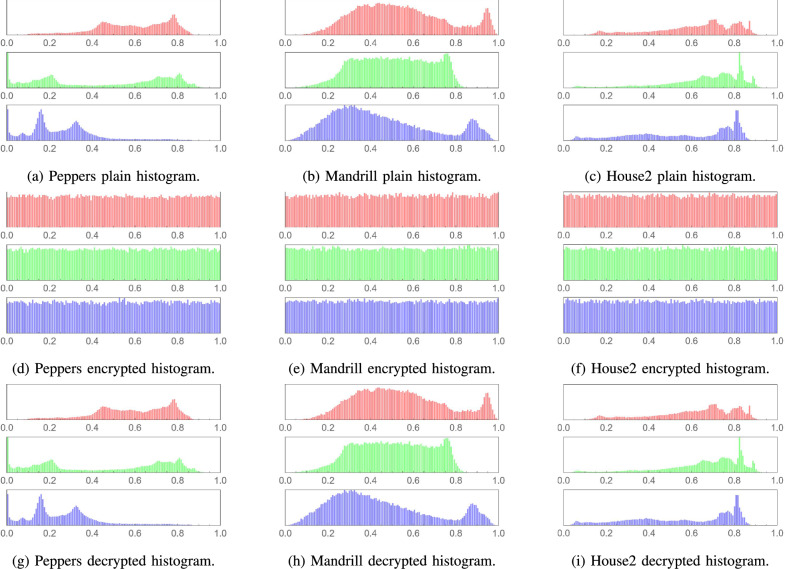
Histogram analysis for various plain images, their encrypted and decrypted versions, respectively.

A histogram analysis is essential for assessing the security of an image encryption algorithm. The histograms of the plain images in this study exhibit distinct patterns reflecting their unique content, as shown in Figs. 10 and 11. After encryption with the proposed algorithm, the histograms of the resulting images were analyzed and found to display a uniform distribution of pixel values.

This uniformity in the encrypted images’ histograms is a key indicator of the algorithm’s effectiveness. It suggests that the encryption process has successfully obfuscated the original patterns and intensity distributions, which is crucial for preventing information leakage. The consistency of uniform histograms across various image types confirms the algorithm’s capability to provide a high level of security regardless of the original image content. Furthermore, the lossless decryption abilities of the proposed algorithm are witnessed in the decrypted images’ histograms, which are identical to those of their plain versions.

### Mean squared error

The Mean Squared Error (MSE) serves as a crucial quantitative metric for evaluating the efficacy of encryption algorithms. The MSE measures the average of the squares of the errors, that is, the average squared difference between the original plaintext image and the encrypted image. Mathematically, it is defined as:

4 $$\begin{aligned} MSE=\frac{\sum _{i=0}^{M-1}\sum _{j=0}^{N-1}(P_{(i,j)}-E_{(i,j)})^{2}}{M\times N}, \end{aligned}$$

where in the context of images with dimensions $M\times N$, the symbols $P_{(i,j)}$ and $E_{(i,j)}$ denote the pixel values at the coordinate (*i*, *j*) in the original and encrypted images, respectively.

A higher MSE value indicates a greater alteration of the original image, which suggests that the encryption algorithm has significantly transformed the image, making it more resistant to various attacks. This is because a well-encrypted image should have little to no resemblance to its original counterpart, leading to a high disparity that the MSE can capture effectively.

The MSE is particularly favored for its sensitivity to changes in pixel values, making it a suitable measure for the strength of encryption. An encryption algorithm that yields a high MSE has likely distributed the pixel intensity changes uniformly across the image, leaving no patterns that could be exploited for decryption without the proper key.

Here, the MSE is employed to compare plain and encrypted images produced by the proposed encryption algorithm. A higher MSE value is indicative of the algorithm’s superior performance in obscuring the original image content, thus enhancing the security of the image data against unauthorized access or deciphering attempts. Table 2 showcases the MSE values for the proposed encryption algorithm alongside those from existing literature. The data reveal that the algorithm’s MSE values are competitive, often exceeding the encryption strength indicated by previously reported metrics, thus affirming the robustness of the proposed algorithm.

**Table 2:** MSE values comparison with the literature.

Images	Proposed	^45^	^46^
Lena	8946.81	8913.31	8917.24
Mandrill	8307.56	8316.4	8322.19
Peppers	10072.5	10144.4	10029.2
House2	9162.74	9172.77	9192.32
Scar	8130.11	*N*/*A*	*N*/*A*
Tissues	10715.3	*N*/*A*	*N*/*A*
Cornea	14005.2	*N*/*A*	*N*/*A*

### Peak signal-to-noise ratio

In evaluating the quality of image encryption, the Peak Signal-to-Noise Ratio (PSNR) is traditionally regarded as a measure where higher values indicate lesser distortion. However, for image encryption applications, especially in the medical imaging domain, this metric is interpreted differently. PSNR is derived from the Mean Squared Error (MSE), which quantifies the variance between pairs of plain and encrypted images. In the context of encryption, an elevated MSE is preferred as it signifies a more secure encryption, demonstrating a greater degree of alteration in the encrypted image from the original.

For the proposed algorithm presented, effectiveness is reflected by the ability to increase the MSE, leading to a lower PSNR that is considered indicative of enhanced encryption strength. This approach, which might seem unconventional, is critical for the security of sensitive medical images by reducing the likelihood of unauthorized information extraction from encrypted images.

The PSNR is mathematically expressed as:

5 $$\begin{aligned} PSNR=10 \log \Big (\frac{I^{2}_{max}}{MSE}\Big ). \end{aligned}$$

Here, $I_{max}$ signifies the highest pixel intensity value that can be found in a grayscale image.

In Table 3, a comparison of PSNR values is provided, contrasting the proposed encryption algorithm with established state-of-the-art methods. It is observed that lower PSNR values are consistently yielded by the proposed algorithm, suggesting superior performance in line with the encryption-centric interpretation of PSNR. These lower PSNR values, as produced by the proposed algorithm, stand as evidence of its robust encryption capabilities, confirming its effectiveness in securing medical images.

**Table 3:** PSNR values of encrypted images comparison with the literature.

Images	Proposed	^45^	^46^	^49^
Lena	8.61412	8.63042	8.6285	8.6503
Mandrill	8.93607	8.93145	8.92843	8.9393
Peppers	8.09945	8.06855	8.11813	8.1446
House2	8.51055	8.5058	8.5058	*N*/*A*
Scar	9.02984	*N*/*A*	*N*/*A*	*N*/*A*
Tissues	7.83076	*N*/*A*	*N*/*A*	*N*/*A*
Cornea	6.6679	*N*/*A*	*N*/*A*	*N*/*A*

### Entropy

In image encryption research, entropy is a critical measure of randomness and security. For color medical images, which typically contain 24-bit pixel values, an entropy value close to 8 for each color channel is considered ideal. This level represents the highest degree of unpredictability in pixel value distribution, making the encrypted image robust against statistical attacks.

The mathematical expression describing the entropy value in each color channel of an encrypted image is given by:

6 $$\begin{aligned} H(m)=\sum _{i=1}^{M} p(m_i) \log _2 \frac{1}{p(m_i)}. \end{aligned}$$

In (6), $p(m_i)$ stands for the likelihood of each pixel value *m* appearing within the entire set of *M* pixel values present in an image.

The proposed algorithm, which is specifically designed for color medical images, has been rigorously tested for its ability to achieve high entropy values. Table 4 compares the entropy of the encrypted images obtained from the proposed algorithm with those from existing algorithms in the literature. It is observed that the values achieved by the proposed algorithm are very close to the ideal value of 8 for each color channel, indicating a near-optimal level of security and randomness.

This performance not only demonstrates the proposed algorithm’s capability in creating highly secure encrypted images but also shows its distinct advantage over some of the counterparts in the literature. The consistently high entropy values close to the ideal mark underscore its suitability for secure medical image encryption applications.

Crucially, the observed entropy values ($\approx 7.998 -- 7.999$) are not merely high; they are found to lie within the sampling fluctuation expected for an ideal uniform source at the tested image sizes: for an image with $M\times N$ pixels per channel, the plug-in estimator is known to exhibit a small negative bias of approximately $(K-1)/(2M\times N ln 2)$ with $K=256$, and a standard deviation that scales as $O(1/M\times N$) yielding a theoretical $95\%$ range near $7.996 -- 8.000$ for images of size $512\times 512$; across all images and channels, The calculated values are observed to fall within this interval, indicating that no statistically significant deviation from ideal behavior is detected, and this conclusion is further supported by consistency across diverse images (high entropy being input-agnostic) and by parity with or improvement over contemporary schemes (Table 4), by which it is shown that histogram flattening and the breaking of low-order redundancies are achieved at state-of-the-art levels.

**Table 4:** Entropy values of encrypted images comparison with the literature.

Images	Proposed	^34^	^45^	^46^	^48^	^32^
Lena	7.999	7.997	7.997	7.999	7.999	7.997
Mandrill	7.999	7.997	*N*/*A*	7.999	7.999	7.997
Peppers	7.999	7.997	*N*/*A*	7.998	7.999	7.999
House2	7.999	*N*/*A*	*N*/*A*	7.999	*N*/*A*	*N*/*A*
Scar	7.998	*N*/*A*	*N*/*A*	*N*/*A*	*N*/*A*	*N*/*A*
Tissues	7.999	*N*/*A*	*N*/*A*	*N*/*A*	*N*/*A*	*N*/*A*
Cornea	7.998	*N*/*A*	*N*/*A*	*N*/*A*	*N*/*A*	*N*/*A*

### Mean absolute error

The Mean Absolute Error (MAE) is a critical metric for assessing the strength of image encryption algorithms. It quantifies the average magnitude of differences between the original and encrypted images without considering the direction of those differences. For robust image encryption, a high MAE value is favorable as it indicates that the encrypted image significantly deviates from the original, making it more secure against attempts to decrypt without the correct key. It is mathematically given by:

7 $$\begin{aligned} MAE=\frac{1}{M\times N}\sum _{i=0}^{M-1}\sum _{j=0}^{N-1}|{P_{(i,j)}-E_{(i,j)}}|. \end{aligned}$$

Within this framework, the pixels of the original image are denoted by $P_{(i,j)}$, while the pixels of the encrypted image are denoted by $E_{(i,j)}$, with each image having a resolution of $M\times N$.

In the context of color medical image encryption, where preserving the confidentiality of the image content is of the utmost importance, the proposed algorithm has been designed to maximize the MAE. The effectiveness of the algorithm in this regard is illustrated in Table 5, which presents the MAE values of the encrypted images. The results show that the MAE values for the proposed algorithm are substantially high, suggesting that the encrypted images bear minimal resemblance to their original counterparts.

**Table 5:** MAE values of encrypted images comparison with the literature.

Images	Proposed	^45^	^46^
Lena	77.7323	77.75407	77.4998
Mandrill	75.082	75.1741	75.2098
Peppers	82.0814	82.3332	81.9145
House2	78.4658	78.5196	78.6348
Scar	74.3994	*N*/*A*	*N*/*A*
Tissues	84.6037	*N*/*A*	*N*/*A*
Cornea	97.3869	*N*/*A*	*N*/*A*

### Pixel cross correlation

Pixel cross-correlation is a key statistical measure used to evaluate the effectiveness of image encryption. In natural images, adjacent pixels typically exhibit high correlation due to similar intensity values. A robust encryption algorithm should significantly reduce this correlation, ideally making it close to zero to prevent statistical inference. Cross-correlation is often measured in horizontal, vertical, and diagonal directions to assess how well spatial relationships are disrupted. Lower values in the encrypted image indicate higher security, as they reflect greater pixel randomness and better concealment of image structure. Thus, pixel cross-correlation is essential for validating resistance to statistical attacks. This metric is expressed mathematically as follows:

8 $$\begin{aligned} \rho (x,y)= \frac{cov(x,y)}{\sqrt{\sigma (x)}\sqrt{\sigma (y)}}, \end{aligned}$$

such that,

9 $$\begin{aligned} cov(x,y)&= \frac{1}{N}\sum _{i=1}^{N}(x_i-\mu (x))(y_i-\mu (y)), \end{aligned}$$

10 $$\begin{aligned} \sigma (x)&= \frac{1}{N}\sum _{i=1}^{N}(x_i-\mu (x))^2, \end{aligned}$$

11 $$\begin{aligned} \mu (x)&= \frac{1}{N}\sum _{i=1}^{N}(x_i). \end{aligned}$$

**Fig. 12:**
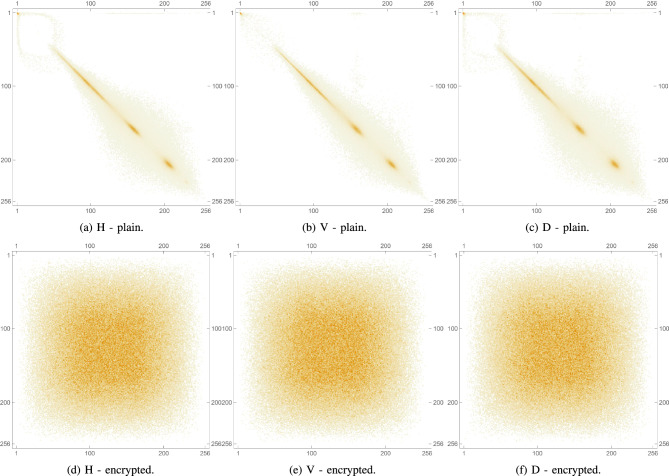
PCC diagrams of plain and encrypted augmented medical images.

**Fig. 13:**
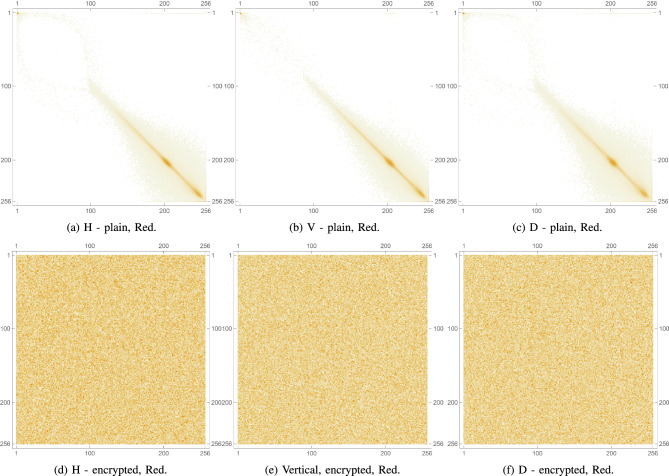
PCC diagrams of the Red channel for plain and encrypted augmented medical images.

**Fig. 14:**
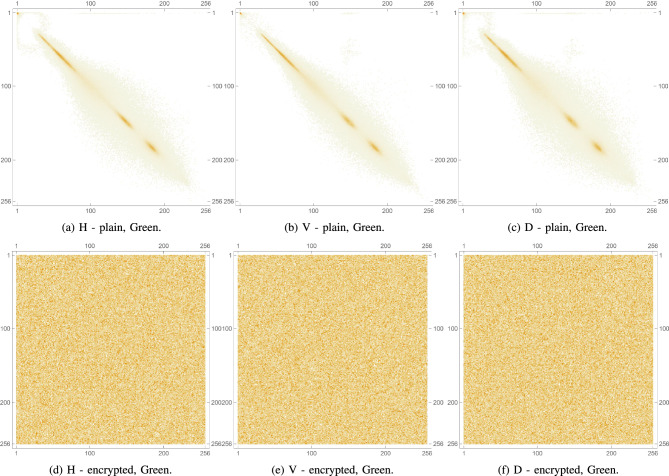
PCC diagrams of the Green channel for plain and encrypted augmented medical images.

**Fig. 15:**
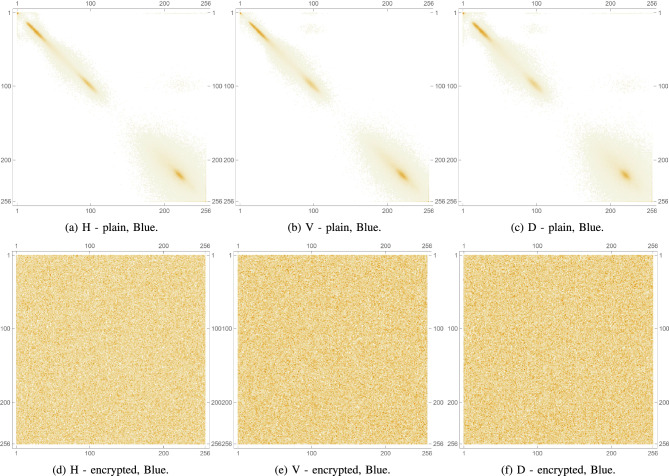
PCC diagrams of the Blue channel for plain and encrypted augmented medical images.

**Fig. 16:**
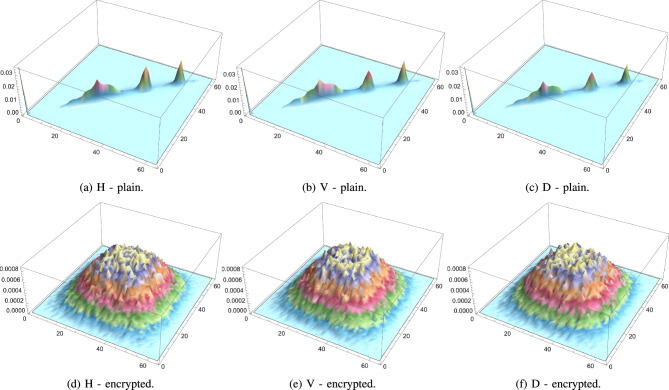
PCC 3D diagrams of plain and encrypted augmented medical images.

For the plain images, as anticipated, the cross-correlation coefficients are relatively high, as shown in Table 6 indicating a strong correlation between adjacent pixels due to the inherent patterns and structures in the images. Conversely, as shown in Table 7, the encrypted images show significantly lower cross-correlation coefficients in all 3 directions, nearing 0. This indicates an effective reduction in predictability and a successful introduction of randomness by the encryption process. These results are visually confirmed by inspecting Fig. 12 which shows the PCC matrix 2D plots for a plain image, as well as for its encrypted version. Furthermore, Figs. 13, 14, and 15 illustrate the same plots, but for the separate RGB color channels of the same plain and encrypted images, while 3D plots are provided in Fig. 16, illustrating the same behavior.

The drastic decrease in PCC in the encrypted images confirms that the proposed algorithm effectively disrupts the spatial relationship between pixels in all directions. This strong disruption of pixel correlation is a crucial indicator of the robustness of the encryption algorithm, which ensures that the encrypted images are secure against statistical attacks that exploit these relationships.

**Table 6:** PCC computed for 3 directions: horizontal, diagonal and vertical on various plain images.

Plain. image	H	D	V
Lena	0.938611	0.913175	0.96833
Peppers	0.959422	0.930426	0.966795
Mandrill	0.848778	0.750624	0.79088
House2	0.907074	0.850781	0.92309
Scar	0.981347	0.964001	0.977929
Tissues	0.715876	0.656161	0.859506
Cornea	0.991024	0.98948	0.997132

**Table 7:** PCC computed for 3 directions: horizontal, diagonal and vertical on various encrypted images.

Enc. image	H	D	V
Lena	0.0034711	0.0113869	$-0.00391109$
Peppers	0.00208076	0.00585174	$-0.00441664$
Mandrill	0.00466137	$-0.00707577$	$-0.00415225$
House2	0.00161219	$-0.00405455$	0.000159458
Scar	0.00214332	$-0.00460903$	$-0.0029245$
Tissues	$-0.00751371$	$-0.00540123$	$-0.0022483$
Cornea	$-0.00354367$	0.00153407	$-0.0081362$

### Discrete fourier transformation analysis

The Discrete Fourier Transform (DFT) is a fundamental tool in the field of signal processing, used to analyze the frequency components of discrete signals. By applying the DFT to an image, which is considered a 2D signal, one can explore its spectral properties. Mathematically, the DFT of an image is expressed as:

12 $$\begin{aligned} F(k,l)=\sum _{i=0}^{N-1} \sum _{j=0}^{M-1} f(i,j) e^{-i 2\pi (\frac{k i}{M}+\frac{l j}{N})}, \end{aligned}$$

where *f*(*i*, *j*) represents the image in the spatial domain, *F*(*k*, *l*) is the image in the frequency domain, and $M\times N$ is the image dimensions.

In the context of a plain image, the DFT would typically show concentrated energy at specific frequencies that correspond to the structural patterns and details within the image. These frequency components are predictable and can potentially reveal information about the image content. However, when an image has been encrypted, its DFT should ideally exhibit a uniform distribution of energy across the frequency spectrum. This homogeneity in the frequency domain indicates that the spatial domain details have been thoroughly disguised, confusing any patterns that could be used to infer the original content of the image.

**Fig. 17:**
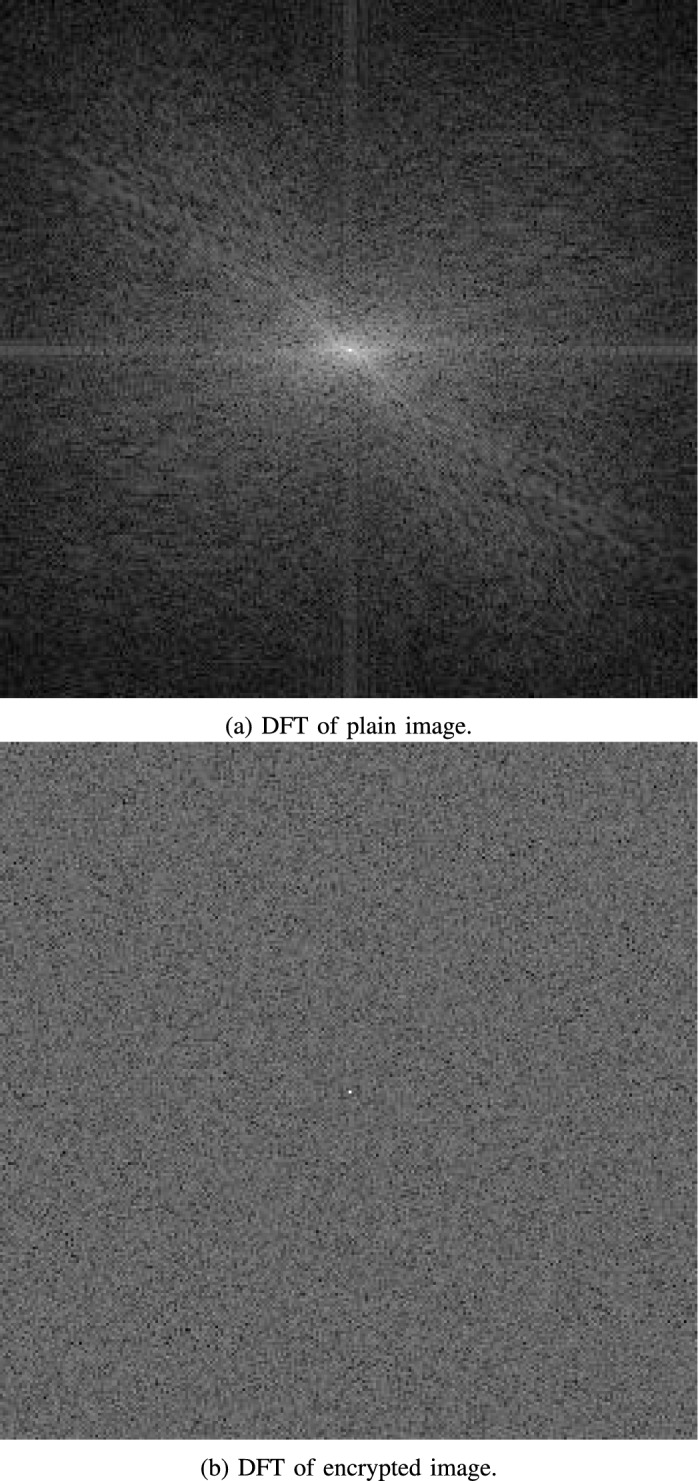
DFT as applied to plain and encrypted image of Lena.

Upon analyzing the DFT of images encrypted with the proposed medical image encryption algorithm, as shown in Fig. 17, a stark contrast is observed when compared to the DFT of their plain counterparts. The encrypted images display a frequency spectrum that lacks any discernible peaks or patterns, demonstrating a highly uniform distribution. This suggests that the spatial domain information is effectively randomized, signifying a successful encryption process.

The DFT analysis thus reinforces the strength of the proposed encryption algorithm. By ensuring that the spectral characteristics of the encrypted image are significantly different from those of the plain image, the algorithm effectively protects the confidentiality and integrity of sensitive medical imagery.

### Differential attack analysis

This section provides an analysis of the proposed algorithm’s resistance to differential attacks. Differential attacks focus on the effect that a single-pixel alteration in the input image has on the encrypted output, and the algorithm’s sensitivity to such perturbations is gauged using 2 statistical metrics: the Number of Pixels Change Rate (NPCR) and the Unified Average Changing Intensity (UACI).

The NPCR is computed using the following expression:

13 $$\begin{aligned} NPCR = \frac{\sum _{i=1}^{M}\sum _{j=1}^{N} D(i,j)}{M \times N} \times 100\%, \end{aligned}$$

where *D*(*i*, *j*) is a binary matrix, such that the value at position (*i*, *j*) is determined by comparing the corresponding pixels at position (*i*, *j*) in the encrypted images before and after the single pixel change in the original image:

14 $$\begin{aligned} D(i,j) = {\left\{ \begin{array}{ll} 1 & \text {if } E_1(i,j) \ne E_2(i,j), \\ 0 & \text {if } E_1(i,j) = E_2(i,j), \end{array}\right. } \end{aligned}$$

while *M* and *N* are the dimensions of the images, representing the number of rows and columns, respectively.

The UACI is determined using the formula:

15 $$\begin{aligned} UACI = \frac{1}{M \times N} \sum _{i=1}^{M}\sum _{j=1}^{N} \frac{\left| P_1(i,j) - P_2(i,j) \right| }{255} \times 100\%. \end{aligned}$$

with $P_1(i,j)$ and $P_2(i,j)$ denoting the pixel values at position (*i*, *j*) in the first and second encrypted images, respectively, and *M* and *N* representing the image dimensions.

The empirical NPCR and UACI, shown in Table 8 for NPCR and Table 9 for UACI, closely match the theoretical optima for 8-bit images (NPCR $\approx 99\%$, UACI $\approx 33\%$), which is the hallmark of diffusion close to an ideal random permutation. In practical cryptanalysis, NPCR above $99\%$ indicates that a single-pixel flip in the plaintext flips almost all ciphertext pixels with probability near 1/2, leaving no exploitable low-diffusion regions. Likewise, UACI near $33\%$ shows that the average ciphertext intensity difference approaches the expectation for two independent uniform images, meaning output differences are distributed as if the cipher were a one-time randomizer rather than a structured transform.

Two additional aspects strengthen this conclusion. First, the small deviation of our NPCR/UACI from the theoretical expectations lies within the sampling variance predicted for images of size $M\times N$, which suggests no systematic bias; residual gaps are statistically insignificant rather than structural weaknesses. Second, the results are consistent across modalities and test images, indicating that diffusion is input-agnostic and not tuned to a specific content distribution.

Together, these observations imply high avalanche strength and effective decorrelation, which are precisely the properties targeted by differential attackers. Under such conditions, differential trails fail to concentrate probability mass, rendering chosen-plaintext perturbations ineffective for key recovery or ciphertext prediction. Consequently, the measured NPCR and UACI provide strong evidence that the proposed scheme resists differential attacks at a level comparable to state-of-the-art image ciphers.

**Table 8:** NPCR values of various encrypted images comparison with the literature.

Images	Prop.	^34^	^45^	^46^	^47^	^48^	^32^
Lena	99.63	99.6	99.7	99.59	99.59	99.67	99.6
Mandrill	99.62	*N*/*A*	*N*/*A*	99.62	*N*/*A*	99.67	99.58
Peppers	99.61	*N*/*A*	*N*/*A*	99.57	*N*/*A*	99.67	99.62
House2	99.62	*N*/*A*	*N*/*A*	99.62	*N*/*A*	*N*/*A*	*N*/*A*
Scar	99.59	*N*/*A*	*N*/*A*	*N*/*A*	*N*/*A*	*N*/*A*	*N*/*A*
Tissues	99.62	*N*/*A*	*N*/*A*	*N*/*A*	*N*/*A*	*N*/*A*	*N*/*A*
Cornea	99.62	*N*/*A*	*N*/*A*	*N*/*A*	*N*/*A*	*N*/*A*	*N*/*A*
Average	99.62	99.6	99.7	99.60	99.59	99.67	99.60

**Table 9:** UACI values of various encrypted images comparison with the literature.

Images	Prop.	^34^	^45^	^46^	^47^	^48^	^32^
Lena	30.48	33.19	30.41	30.39	33.59	33.26	30.72
Mandrill	29.44	*N*/*A*	*N*/*A*	29.49	*N*/*A*	33.62	27.84
Peppers	32.19	*N*/*A*	*N*/*A*	32.12	*N*/*A*	33.55	31.05
House2	30.77	*N*/*A*	*N*/*A*	30.83	*N*/*A*	*N*/*A*	*N*/*A*
Scar	29.17	*N*/*A*	*N*/*A*	*N*/*A*	*N*/*A*	*N*/*A*	*N*/*A*
Tissues	33.17	*N*/*A*	*N*/*A*	*N*/*A*	*N*/*A*	*N*/*A*	*N*/*A*
Cornea	38.19	*N*/*A*	*N*/*A*	*N*/*A*	*N*/*A*	*N*/*A*	*N*/*A*
Average	31.92	33.19	30.41	30.71	33.59	33.48	29.87

### Occlusion and noise attack analysis

The robustness of the proposed image encryption algorithm against noise attacks is thoroughly investigated in this section. Specifically, the algorithm’s resilience to salt-and-pepper (S&P) noise and Gaussian noise is evaluated. Encrypted medical images are subjected to varying levels of these noise types to simulate real-world scenarios where transmitted data may be corrupted by noise.

**Fig. 18:**
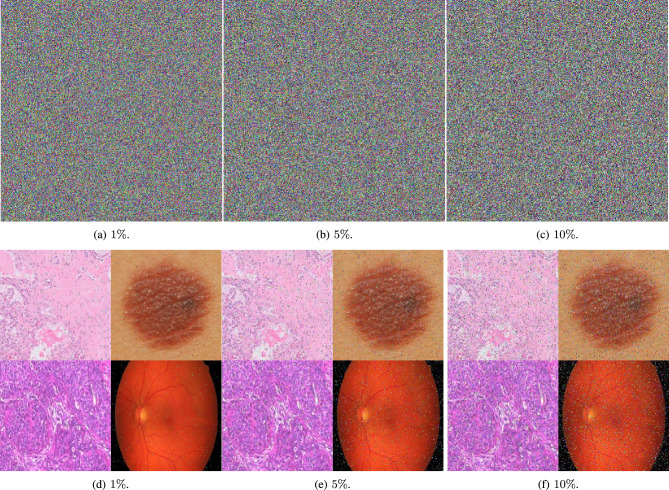
A visual representation of salt and pepper attacks with different intensities on medical augmented images.

**Fig. 19:**
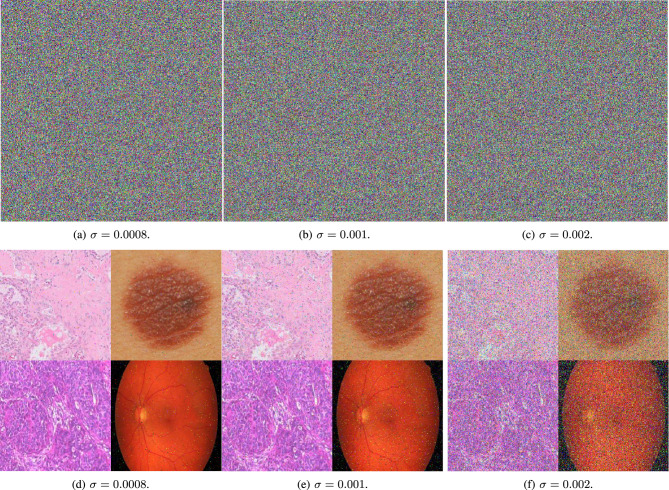
A visual representation of Gaussian noise attacks with different intensities on medical augmented images.

Figure 18 illustrates the encrypted images after the addition of S&P noise at different percentages are presented. It serves to demonstrate the encryption algorithm’s ability to withstand noise interference. In addition to S&P noise, the encrypted images are also subjected to Gaussian noise of various intensities. Fig. 19 provides a visual representation of the algorithm’s performance when faced with this type of statistical noise.

The decrypted images, which are obtained after applying the decryption process to the noise-corrupted encrypted images, are also showcased in Fig. 18 and Fig. 19. Despite the presence of noise, the decrypted images appear to be only slightly affected when compared to their original, plain counterparts. This subtle impact on the decrypted images highlights the encryption algorithm’s capability to maintain the integrity of the original information in the presence of noise, thereby confirming its robustness against such attacks. The visual quality of the decrypted images, alongside the quantitative analysis, suggests that the proposed algorithm is well-suited for practical applications where encrypted images may be subject to noise.

**Fig. 20:**
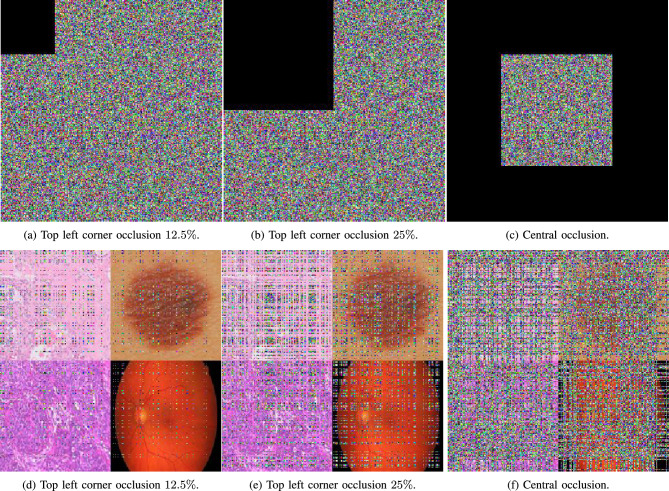
A visual representation of occlusion attacks with different intensities on medical augmented images.

While the resilience of the proposed image encryption algorithm to occlusion attacks is demonstrated in Fig. 20, where a set of encrypted images that undergo partial occlusion, followed by decryption, are depicted. Despite the occlusion, the decrypted images exhibit a degree of recognizability.

The visual results, as depicted, confirm that the proposed algorithm effectively recovers information from occluded encrypted images. The integrity of the visual content is sufficiently maintained, showcasing the algorithm’s capacity to handle data loss scenarios. Figure 20 serves as evidence of the algorithm’s robustness against occlusion, ensuring reliable decryption in adverse conditions.

**Fig. 21:**
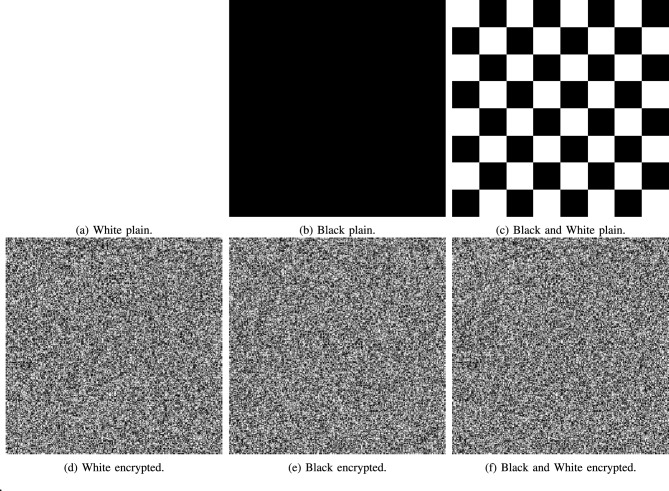
A visual representation of chosen-plaintext attacks.

### NIST SP 800-22 analysis

In this subsection, a comprehensive analysis of the security properties of the proposed algorithm is presented. The robustness of the encrypted output bitstream is rigorously evaluated by subjecting it to the battery of statistical tests defined in the NIST Special Publication 800-22 suite, which is a recognized standard for assessing the randomness of binary sequences essential for the strength of encryption algorithms. For each of the tests, a bit-stream of length $10^6$ resulting from the concatenation of multiple output encrypted augmented images, is utilized. Detailed in Table 10 are the results, indicating that the bitstream generated by the algorithm passed all the tests in the suite successfully (by having *p*-values greater than 0.01). This denotes that a high degree of randomness, indicative of effective encryption, is exhibited by the bitstream, suggesting that the algorithm is equipped with resilience against a variety of statistical attacks and reinforcing its suitability for secure cryptographic applications.

**Table 10:** Results of the NIST suite of tests.

Test	*p*-value	Conclusion
Frequency	0.846176	Success
Block Frequency	0.299220	Success
Run	0.611480	Success
Long runs of ones	0.603139	Success
Rank	0.709478	Success
Spectral FFT	0.266839	Success
Non overlapping	0.717107	Success
Overlapping	0.695607	Success
Universal	0.735293	Success
Serial	0.623364	Success
Serial	0.899439	Success
Approx. entropy	0.713425	Success
Cum. sums forward	0.652234	Success
Cum. sums reverse	0.483901	Success
Random Excursions (RE) 1	0.887155	Success
RE 2	0.927176	Success
RE 3	0.789477	Success
RE 4	0.962458	Success
RE 5	0.789979	Success
RE 6	0.864265	Success
RE 7	0.943795	Success
RE 8	0.824661	Success
Random Excursions Variant (REV) 1	0.113316	Success
REV 2	0.065541	Success
REV 3	0.088335	Success
REV 4	0.117353	Success
REV 5	0.077089	Success
REV 6	0.221157	Success
REV 7	0.986949	Success
REV 8	0.751429	Success
REV 9	0.977206	Success
REV 10	1.000000	Success
REV 11	0.621466	Success
REV 12	0.832754	Success
REV 13	0.928315	Success
REV 14	0.972430	Success
REV 15	0.574914	Success
REV 16	0.279810	Success
REV 17	0.133263	Success
REV 18	0.086518	Success

### Key space analysis

Key space refers to the total number of unique keys that can be used in an encryption algorithm. A large key space is critical for enhancing security as it directly translates to increased resistance against brute-force attacks, where an attacker attempts to decode the encrypted data by systematically trying every possible key.

For the proposed color medical image encryption algorithm, the key space has been meticulously engineered to be as large as possible. This ensures that the number of potential keys is vast, making brute-force attacks impractically time-consuming and computationally expensive. The choice of the hyperchaotic system in (3) allows for the effective utilization of 37 parameters. Moreover, the Gold sequence is initialized with a single seed. This means that there is a total of 38 parameters. For a machine with a precision of $10^{-16}$, this leads to a key space of $10^{38 \times 16}=10^{608}\approx 2^{2020}$. This attained key space of $2^{2020}$ is above the threshold suggested in earlier research of $2^{100}$ and deemed sufficient for deterring brute-force attacks^59^.

As indicated in Table 11, the key space of the proposed algorithm is quantitatively analyzed and compared with the key spaces of other encryption methods in the current literature. The proposed algorithm exhibits a significantly larger key space, which implies a superior level of security, with the only exception being that of the algorithm proposed in^45^. The expansive key space of the proposed algorithm not only deters the feasibility of brute-force attacks but also places it at the forefront of encryption techniques where safeguarding medical image data is paramount.

**Table 11:** Key space comparison with the literature.

Algorithm	Key space
Proposed	${10^{608}}\approx 2^{2020}$
^34^	$2^{320}$
^45^	$2^{52141}$
^46^	$2^{1754}$
^47^	$2^{256}$
^50^	$2^{35}$
^60^	$2^{541}$
^61^	$2^{260}$
^62^	$2^{287}$

### Runtime analysis

The runtime performance of the proposed image encryption algorithm is meticulously examined in this section. Table 12 is referenced, which exhibits the encryption times for images across a range of dimensions, offering a comprehensive perspective on the processing efficiency for various image sizes. Tables 13 and 14 describe the detailed encryption and decryption time stages for the proposed scheme. Moreover, Table 15 is brought to attention, comparing the encryption time of a $256\times 256$ image using the proposed algorithm against those reported in the literature.

It is observed that the proposed algorithm demonstrates superior code efficiency in comparison to most of the existing algorithms, as detailed in Table 12. This enhanced performance is largely attributed to the employment of parallel processing techniques, along with the strategy of batch encrypting medical images by augmenting several into a single image. The encryption time of a $256\times 256$ image translates into an encryption rate of 15.5 Mbps. Furthermore, upon comparing the encryption time of the proposed algorithm with that of a lightweight counterpart, the ASCON algorithm, the proposed algorithm is shown to exhibit a much lower encryption time.

The exception noted is the algorithm proposed by Kumari et al. in^32^, which records shorter encryption times. Their algorithm is reported to have been implemented on computing resources that are significantly more advanced, a factor that likely contributes to the exceptionally short encryption times they have attained. This consideration is vital in justifying the comparative analysis and underscores the importance of contextualizing the algorithm’s runtime within the scope of available computational capabilities. (Fig. 21).

**Table 12:** Encryption time of an augmented medical image at various dimensions.

Augmented image dimensions	Time [s]
$128 \times 128$	0.190679
$256 \times 256$	0.406277
$512\times 512$	0.998701
$1024\times 1024$	4.29191

**Table 13:** Encryption time of the proposed scheme.

Operation	Time [s]
XOR time	0.0092605
Moore’s Automation	0.320886
Sbox	0.0057435
Total	0.33589

**Table 14:** Decryption time of the proposed scheme.

Operation	Time [s]
XOR time	0.0073362
Moore’s Automation	0.308162
Sbox	0.0061134
Total	0.321611

**Table 15:** Time analysis of encrypting an image of dimensions $256 \times 256$ comparison with the literature.

Scheme	Time [s]	Computer Specs
Proposed	0.406277	AMD^®^ Ryzen 5 5600*H*, 3.3 GHz, 16 GB
Ascon (3 variants)^63^	4.57441	N/A
^34^	8.639131	Intel^®^ Core^TM^ i$7-8550$U CPU @ 1.80 GHz, 8 GB
^45^	0.427	AMD^®^ Ryzen^TM^ 5600H Mobile 3.3 GHz, 16 GB
^46^	0.021658	3.3 GHz AMD^®^ Ryzen 9 5900HX, 32GB
^47^	0.473	Intel^®^ Core^TM^ i$7-6700$ CPU @ 3.40 GHz, 8 GB
^32^	0.006686	Intel Xeon I^®^ CPU E$5-1620$, 3.5 GHz, 64 GB

### Memory consumption analysis

**Fig. 22:**
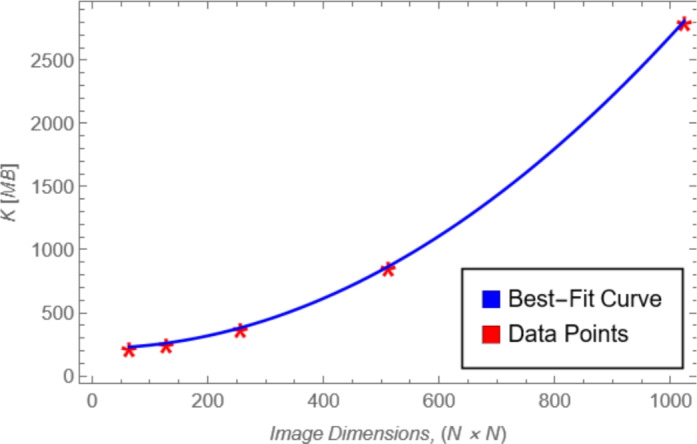
Memory consumption behavior.

Figure 22 illustrates the memory consumption of the augmented image as a function of its dimensions. The results demonstrate that memory usage increases linearly with the image size, starting at approximately 230 MB for a $64 \times 64$ image and rising to approximately 2810 MB for a $1024 \times 1024$ image. This linear scaling indicates that the proposed algorithm maintains predictable and manageable memory requirements, supporting its computational efficiency even for larger image dimensions. Moreover, Table 17 shows how increasing the number of iterations *N* affects the memory usage.

### Big O complexity

In relation to the proposed algorithm’s complexity in terms of the Big O notation, it has a complexity of $O(M \times N)$, where *M* and *N* represent the dimensions of an image. Table 16 shows a comparison of the proposed algorithm’s complexity with its counterparts from the literature. It is clear that the various algorithms’ complexity are comparable.

**Table 16:** Algorithm complexity in terms of the Big O notation.

Scheme	Time complexity
Proposed	$O(M\times N)$
^34^	$O(M^3)$
^47^	$O(N^2)$

### Iteration analysis

By running the algorithm *N* times over an image, one could investigate to what extent the security performance improves, as well as the effect on memory requirements and complexity. Table 17 shows how increasing the number of iterations *N* affects performance, in terms of MSE and entropy values.

**Table 17:** Effect of number of iterations *N* on the security performance, in terms of MSE and entropy, as well as memory usage, and encryption time.

*N*	Image	MSE	Entropy	Memory [MB]	$T_{Enc}$
1	Lena	8886.96	7.99882		
	Mandrill	8285.69	7.9989		
	Peppers	10127.3	7.99909	864.648	0.406277
	House2	9162.05	7.99901		
3	Lena	8946.81	7.99908		
	Mandrill	8307.56	7.99907		
	Peppers	10072.5	7.99905	904.34	0.896185
	House2	9162.74	7.99904		
5	Lena	8934.82	7.99901		
	Mandrill	8269.0	7.99911		
	Peppers	10047.7	7.99905	959.967	1.50765
	House2	9169.99	7.99906		

### S-box performance analysis

Substitution boxes (S-boxes) are pivotal elements in cryptographic algorithms, serving as the core component in non-linear transformation of inputs into outputs. The efficacy of an S-Box as a part of an encryption algorithm can be evaluated through several critical tests, each measuring different aspects of its performance:

1. Non-linearity (NL): This metric examines how closely an S-Box’s performance can be approximated by affine functions, which are linear combinations of the input bits^64^. A Boolean function’s truth table is scrutinized to determine the minimum number of bit alterations required to convert the function into its nearest affine counterpart. High NL is essential as it ensures that the relationship between the plaintext and the ciphertext is complex, thereby providing robustness against linear cryptanalysis.
2. Linear Approximation Probability (LAP): The LAP test calculates the probability of an S-Box exhibiting linear behavior, which is an undesirable property in encryption as it can lead to biases exploitable by attackers^65^. An effective S-Box should have a low LAP, indicating that it is resistant to linear attacks and thus enhances the security of the encryption algorithm.
3. Differential Approximation Probability (DAP): This measure evaluates the impact of specific alterations in the input bits on the resulting output bits^66^. It seeks to quantify the predictability of changes in the output when known changes are applied to the input. A robust S-Box should have a low DAP, indicating that it provides strong protection against differential cryptanalysis by ensuring that input changes do not lead to discernible patterns in the output.
4. Bit Independence Criterion (BIC): This criterion assesses the extent to which the changes in different input bits contribute independently to the output bits^66^. An ideal S-Box would cause each output bit to change independently in response to an alteration of a single input bit, thereby eliminating pattern formation in the ciphertext that could be statistically correlated to the plaintext.
5. Strict Avalanche Criterion (SAC): The SAC test measures how effectively a change in a single input bit affects each bit of the output^66^. For robust encryption, each output bit should have a $50\%$ chance of changing whenever a single input bit is flipped, ensuring that the output exhibits an avalanche effect. This means that a minimal change in the input leads to a significant and unpredictable change in the output.

Table 18 shows the achieved values of the above-mentioned metrics as the proposed encryption scheme’s S-Box is subjected to them to validate its effectiveness and security. It is demonstrated that the proposed S-box design adheres to these criteria, ensuring a high level of security for encrypted medical images. Furthermore, the same table compares the achieved values of the metrics with those reported in the literature for counterpart image encryption algorithms. It is demonstrated that the proposed algorithm exhibits comparable security performance.

**Table 18:** Comparison between the proposed S-boxes and those in the literature.

S-Box	NL	SAC	BIC	LAP	DAP
Proposed S-box	108	0.50147	108	0.07812	0.01562
^45^	108	0.49414	108	0.07812	0.01562
^46^	110	0.50073	108	0.07812	0.01562
^47^	112	0.5	*N*/*A*	*N*/*A*	*N*/*A*

### Chi square

Chi square $\chi ^2$ is a statistical analysis used to assess the randomness and uniformity of pixel values in an encrypted image. It is described as follows:

16 $$\begin{aligned} \chi ^2&=\sum _{i=0}^{255}\frac{(O_i-E_i)^2}{E_i}, \nonumber \\ E_i&=\frac{N\times M}{256}, \end{aligned}$$

where $O_i$ represents the observed frequency and $E_i$ the expected frequency. Table 19 shows the values of Chi square with encrypted images.

**Table 19:** Chi Square test on various images.

Image	$\chi ^2$	Conclusion
Peppers	260.03	Success
Mandrill	257.755	Success
House2	260.719	Success
Tissue	258.356	Success
Cornea	255.996	Success
Scar	255.543	Success

The computed $\chi ^2$ values in Table 19 are close to the ideal value of 293.247. This indicates that the pixel distribution in the encrypted images is nearly uniform, confirming a high level of encryption quality. The slight deviations are expected and acceptable, showing that the encryption method effectively obscures any recognizable patterns in the original images. Overall, the resulting encrypted images from the proposed algorithm reflect robust and effective encryption.

### Resilience against chosen-plaintext, chosen-ciphertext, and side-channel attacks

In a chosen-plaintext attack (CPA), the adversary can submit any plain images they like and receive the corresponding ciphertexts. Their aim is to recover the key or infer details about the encryption method. This model assumes the attacker fully understands the cryptosystem’s design. Even so, without the secret keys, the enormous key space of the proposed scheme makes a successful attack infeasible. As shown in Subsection "Key space analysis", the key space is $2^{2020}$.

Conversely, in a chosen-ciphertext attack (CCA), the attacker can pick arbitrary ciphertext images and obtain their decrypted plaintexts. CCAs are especially pertinent to public-key systems, but that is not applicable here because the proposed image encryption uses symmetric keys as in Fig. 21.

Side-channel attacks rely on stable, data- or key-dependent variations across many traces, but the proposed symmetric scheme deprives them of useful signal: ciphertexts and intermediates are effectively noise-like (near-ideal entropy, uniform histograms, near-zero PCC, flat DFT) with strong diffusion (NPCR $\approx 99.6\%$, UACI $\approx 31.9\%$) that quickly destroys locality, while processing is linear-time, branchless, and input-agnostic (XOR and Moore automaton constant-time; S-box key-derived and implementable via bitslicing/masked lookups), so timing/cache patterns don’t correlate with content. Per-session keys and independently seeded MCNNM/Gold components prevent cross-run trace aggregation; the high-dimensional hyperchaotic keystream is extremely sensitive to parameters, impeding template/recovery from coarse power/timing features; and the symmetric deployment without a public decryption oracle (with AEAD, encrypt-then-authenticate) blocks adaptive leakage amplification. Together, these properties render side channels statistically uninformative and practically ineffective against the proposed software implementation.

### Deep learning–based cryptanalysis

Residual structure, correlations, or oracle leakage are typically exploited by generative attackers^67^. In the present work, ciphertexts are shown to be statistically indistinguishable from noise, as evidenced by passing the NIST SP 800–22 battery in Table 10, by achieving near-ideal entropy (approximately 7.998 to 7.999) in Table 4, by exhibiting uniform histograms in Figs. 10 and 11, by attaining near-zero pixel cross-correlation in Table 7 with corresponding 2D/3D PCC plots in Figs. 12, 13, 14, 15, 16, and by presenting a uniform DFT magnitude in Fig. 17. Strong diffusion and nonlinearity are provided by the MCNNM-derived keystreams, key-dependent Gold-sequence S-boxes, and Moore-automaton confusion, with NPCR and UACI reported in Tables 8 and 9 (approximately $99.6\%$ and $31.9\%$, respectively), so that low-order cues leveraged by data-driven models are removed. While impossibility results against arbitrary black-box learners are not claimed, the ciphertext-only setting is characterized by a distribution approaching uniform noise, and in known-plaintext settings cross-session generalization is hindered by per-session keys and nonces together with heavy diffusion. For deployment, encrypt-then-authenticate is recommended via authenticated encryption with associated data (AEAD) so that decryption-oracle abuse is eliminated, per-session keying is encouraged so that dataset aggregation is prevented, and constant-time, branchless implementations are advised so that timing and cache side channels are mitigated.

## Conclusions and future outlook

The research conducted in this study culminated in the development of a sophisticated encryption algorithm that is tailored for the secure transmission of medical images through cloud services. In the initial phase, the consolidation of multiple images into a single augmented image, followed by a first layer of encryption using an S-box generated by a Memristive Coupled Neural Network Model (MCNNM), was successfully implemented. The subsequent integration of Moore’s Automaton with Gold sequences as a confusion mechanism proved effective in scrambling the image structure, thus mitigating pixel correlations. The iterative process over *N* cycles was shown to be not significant in enhancing the depth, and thus, the performance of encryption. The extensive evaluations underscored the considerable key space ($2^{2020}$) and high software encryption rate (15.5 Mbps) of the proposed algorithm. Furthermore, the algorithm withstood rigorous statistical tests, confirming its resilience and marking it as a highly secure method for the protection of sensitive healthcare data. However, a limitation of this work is that it provides no integrity or tamper detection for the resulting encrypted images.

For future research, there is a compelling opportunity to integrate the current encryption methodology with blockchain technology, which could revolutionize the security and traceability of medical image and video transmission within cloud services^68,69^. Additionally, exploring hardware implementation could significantly accelerate the encryption process and enhance security by minimizing software-based vulnerabilities, as in^70,71^. Lastly, employing machine learning techniques to refine the key generation process could yield a more adaptive and robust encryption framework, potentially leading to improvements in both security and performance^10,72^. Moreover, to future-proof deployments, we will increase effective keying and employ post-quantum key encapsulation mechanisms (KEMs) for session establishment, combined with authenticated encryption with associated data (AEAD) for integrity and oracle resistance; and we will extend the algorithm to volumetric (3D) and dynamic (4D) imaging via voxel-block/slice-tile processing with inter-slice decorrelation and GPU parallelism to sustain near real-time clinical workflows. These advancements could collectively elevate the practical applicability and resilience of the proposed encryption system in the healthcare domain.

## Data Availability

The utilized image datasets in this work are obtained from: 1) https://sites.google.com/site/aacruzr/image-datasets 2) http://sipi.usc.edu/database/
